# In the I of the beholder: an attempt to capture the implicit self-concept regarding psychopathy

**DOI:** 10.3389/fpsyg.2024.1346029

**Published:** 2024-06-17

**Authors:** Jonas Krüppel, Dahlnym Yoon, Katharina Zerres, Franziska Brunner, Andreas Mokros

**Affiliations:** ^1^Chair of Personality Psychology, Legal Psychology, and Assessment, Faculty of Psychology, FernUniversität in Hagen, Hagen, Germany; ^2^Institute for Forensic Psychology & Forensic Medicine, Medical School Hamburg (MSH), Hamburg, Germany; ^3^Institute for Sex Research, Sexual Medicine and Forensic Psychiatry, University Medical Center Hamburg-Eppendorf, Hamburg, Germany

**Keywords:** psychopathy, self-concept, insight, indirect measures, Implicit Association Test

## Abstract

This article explores the implicit self-concept pertaining to psychopathy. Two online studies showed inconsistent results, with Study 1 (*n* = 243) suggesting that psychopathy is linked to an implicit self-concept marked by low empathy and Study 2 (*n* = 230) implying no such relationship. In a sample of offenders and community controls (Study 3a, *n* = 166), higher scores on the *Psychopathy Checklist-Revised* (PCL-R) were related to an implicit self-concept of being less rather than more antisocial, and the implicit self-concept showed incremental validity compared to the explicit self-concept. The retesting of an offender subsample (Study 3b, *n* = 47) yielded no evidence for temporal stability or convergent validity. The implicit self-concept of highly psychopathic individuals thus appears to vary, depending on the social context. Future studies should replicate these results in different samples, using additional external correlates.

## Introduction

Psychopathy is one of the central criminogenic factors addressed in forensic psychology and related to various manifestations of antisociality, including violent conduct (Blais et al., 2014), future delinquency (Hanson and Morton-Bourgon, 2005), and institutional misbehavior (Olver et al., 2020). According to one influential description, the construct itself comprises four distinct aspects: Deceitfulness, lack of empathy, impulsivity, and a proneness for antisocial behavior. In correctional settings, these four facets are often assessed through an observer-rating instrument, the *Hare Psychopathy Checklist-Revised* (PCL-R; Hare, 2003; for a review see Hare et al., 2013). Even though different authors have criticized the PCL-R model for its focus on antisocial behavior and therefore proposed alternatives (e.g., Patrick et al., 2009), the measure itself remains one of the dominant assessment instruments with correctional samples (see Hollerbach et al., 2020).

Traditionally, psychopathy has been considered a personality disorder, either in its own right or as a variant of Antisocial Personality Disorder (Coid and Ullrich, 2010). More recently, sub-clinical manifestations have been incorporated in the Dark Tetrad (Paulhus, 2014). This notion of a continuum of psychopathic trait levels covering the full range from mild to most severe forms is concomitant with taxometric research (see, e.g., Mokros et al., 2020, for an overview).

So far, relatively few studies addressed the degree of convergence between observer-ratings of highly psychopathic individuals and their own self-concept regarding the aforementioned personality traits (e.g., lack of empathy, antisociality). In this study, the term personality self-concept is used to describe an individual’s knowledge about his or her personality traits (see Schnabel et al., 2008). Gathering more information about the self-concept regarding psychopathic personality traits can be of huge practical importance, as these traits have been linked to various types of antisocial behaviors (Sellbom et al., 2018). Furthermore, a lack of insight into one’s individual deficits is associated with a lower probability of treatment success (see Sewall and Olver, 2019).

So far, the extant studies yielded moderate to high convergence between self-and informant ratings on psychopathic traits (*r* ≥ 0.34, Miller et al., 2011, 2014; Kelley et al., 2018). Based on these findings, the self-image of highly psychopathic individuals is largely in line with how other people see them apparently. More specifically, highly psychopathic individuals tend to view themselves as, for instance, relatively callous, antagonistic, antisocial (Miller et al., 2011, 2014), and mean (Kelley et al., 2018). These findings are at odds with long-standing speculation that highly psychopathic individuals might be unable to describe themselves in a realistic way (e.g., Lilienfeld, 1994; Sellbom et al., 2018). As a reviewer pointed out, however, they were also either based on the TriPM model (Patrick et al., 2009), a debated psychopathy model (see Sleep et al., 2019), or drawn from community samples, or both.

Furthermore, constructs are often more comprehensively captured when more than one indicator is used (see also Lilienfeld, 1994). Various studies showed that additional variance is explained when behavior is predicted based on both deliberate and automatic self-perceptions (e.g., Schnabel et al., 2006; Back et al., 2009). In the case of psychopathy, it seems plausible to assume that the affective deficits, in particular, are not covered adequately by deliberate self-description (Lilienfeld, 1994; Sellbom et al., 2018). Indeed, recent research implies that highly psychopathic participants have deficits in the automatic processing of emotional stimuli but can intentionally dissimulate their deficits in empathy (Meffert et al., 2013) or perspective taking (Drayton et al., 2018) when being instructed to do so. This disparity between automatic and controlled processing might be reflected by dissociations between the so called explicit and implicit personality self-concept.

When using the controversial term *implicit* (for an overview of the debate, see Greenwald and Lai, 2020), we refer to the definition by De Houwer et al. (2009) who propose that a construct can be labeled as implicit to the degree that the underlying process is automatic (e.g., unintentional, fast; *cf.* de Houwer et al., 2009). Accordingly, the implicit personality self-concept can be described as self-knowledge about one’s personality that is activated automatically, and the explicit personality self-concept as the respective knowledge activated by controlled processes (for similar definitions, see Teige et al., 2004; Schnabel and Asendorpf, 2010).

Implicit mental constructs are commonly assessed through *indirect measures*, a class of instruments that focus on observed rather than self-reported behavior (De Houwer, 2006). The implementation of these measures was propelled by research using the *Implicit Association Test* (IAT; Greenwald et al., 1998). In the IAT, the dependent variable is measured in terms of reaction time (RT) differences between experimental blocks in which two attributes and two categories are combined in either one way (e.g., Luke Skywalker and good, Darth Vader and bad) or the other (e.g., Luke Skywalker and bad, Darth Vader and good). The IAT is based on the idea that individuals respond faster when strongly associated stimuli are paired compared to couplings of weakly associated stimuli (Schnabel et al., 2008). Individuals with an extraverted self-concept, for instance, should thus respond faster when *me* is combined with *extraverted* compared to trials in which *me* is combined with *introverted* (*c.f.* Back et al., 2009). Although IAT-measures mostly used words to represent the different categories, some studies combined words and pictures (e.g., Karpinski and Steinman, 2006).

The IAT was successfully used in countless studies (see Greenwald and Lai, 2020) and modified for different purposes, including the assessment of attitudes towards just one category via the *Single-Category* Implicit Association Test (SC-IAT; Karpinski and Steinman, 2006). At the same time, it has been criticized for several reasons, including the persisting confusion about the underlying process as well as a potential lack of construct validity (see, e.g., Meissner et al., 2019; Schimmack, 2021). Even critics, however, highlighted the IATs potential as an additional measure that might help to reduce measurement error resulting from the sole use of self-reports (e.g., Schimmack, 2021).

In the domain of forensic psychology, predictive validity of indirect self-concept measures has been established especially regarding criminal behavior (e.g., Rivera and Veysey, 2018). With respect to psychopathy, discrepancies between the automatic and controlled self-concept might explain why highly psychopathic individuals are less likely to abstain from (re-)offending (Hanson and Morton-Bourgon, 2005; Forsman et al., 2010) even though they allegedly know about their deficits. To the best of our knowledge, however, only very few studies so far investigated the relationship between psychopathy and the implicit self-concept (for other work on psychopathy and implicit cognition, see, e.g., Snowden et al., 2004). Using the IAT or the SC-IAT, these studies yielded heterogenous results that suggest a self-concept of being similarly dominant and guilty compared to others (Nentjes et al., 2017), and being either more (Suter et al., 2017) or less inclined to break formal rules (Suter et al., 2014). A recent study by Pink et al. (2023), in addition, successfully linked psychopathy according to the TriPM Model to a self-concept of being rather bold, mean, and disinhibited. This study, however, was conducted in a community sample, using self-report measures for a less established psychopathy model.

Thus, it seems timely and reasonable to further explore the validity of indirect self-concept measures for the assessment of psychopathy. First, because the current findings are contradictory. Second, because such measures could provide a glimpse into how highly psychopathic individuals view themselves implicitly. Complementing the existing assessment options with indirect measures could reveal additional features of the disorder and resolve problems of the current self-and observer-report measures (e.g., Boccaccini et al., 2014; Sellbom et al., 2018) by being less time-consuming and reducing the probability of observer effects and dissimulation (Schmidt et al., 2015).

### The present research

Given the reliance of the customary IAT on two opposing categories (such as violence and peace), different authors stressed the need for alternatives to the IAT regarding concepts that lack a natural opposite (e.g., De Cuyper et al., 2017) – a claim that appears particularly relevant with respect to the concept of the *self*. After all, it is unclear what the opposite of *self* is: Other people, non-self, non-existence, or something completely different. Therefore, we decided to abstain from using a control condition and adapted the SC-IAT for the separate assessment of the implicit self-concept regarding (a) empathy and (b) antisocial behavior. Compared to the other aforementioned features (i.e., deceitfulness, impulsivity), these two have often been highlighted as defining features of psychopathy (e.g., Brook et al., 2013).

The corresponding measures were tested in four studies, conducted with correctional and community samples. We decided to measure psychopathy according to the PCL-R model as it affords the possibility to assess the construct in both populations. This model was derived from observer rating instruments but works equally well for corresponding self-report instruments (Neumann et al., 2015). Convergent validity was therefore estimated using the *Self-Report Psychopathy Scale, 4th edition* (SRP 4; Paulhus et al., 2016), the PCL-R or its *Screening Version* (PCL:SV; Hart et al., 1995), and measures for conceptually related traits and behaviors.

As they more often show antisocial and less often show empathic behaviors (Hare, 2003), highly psychopathic individuals were expected to ascribe both types of behaviors more strongly to themselves than to others. They were thus assumed to have a self-concept of being less empathic and more antisocial than their peers. All four study protocols were approved by either the ethics committee of the German Psychological Association (DGPs), the local ethics representative/ethics committee of the host university, or both. Preregistration forms and anonymized data sets^1^ for all four studies can be retrieved from the Open Science Framework^2^.

## Study 1

Study 1 was a first test of the aforementioned relationships in an online-sample. As low SC-IAT scores imply a self-concept of being less empathic and more antisocial, respectively, negative relationships were expected between both SC-IATs and the SRP 4 total score (H1). With regard to the SRP 4 subscales, the *empathy SC-IAT* (eSC-IAT) scores were expected to be negatively related to the Affective and Interpersonal scales of the SRP 4 (H1.1 and H1.2). Similarly, negative relationships were expected between the *antisociality SC-IAT* (aSC-IAT) and the Lifestyle as well as the Antisociality scale of the SRP 4 (H1.3 and H1.4)^3^.

### Materials and methods

#### Participants

*A-priori* sample size estimation was conducted using G*Power 3.1.9.2 (Faul et al., 2009), resulting in a minimum sample size of 67 individuals in order to identify a medium sized correlation coefficient (*r* = 0.30) with a statistical power of 80% at a type I error rate of 5%. Complete data sets were collected from 243 subjects (79% female), recruited via university websites, e-mail, and social media. The final sample consisted of 227 participants (80% female, *M*_age_ = 32.22, *SD*_age_ = 10.85; see Supplementary material for information on sample characteristics, preprocessing, and exclusions).

#### Instruments

Both domains of the implicit self-concept were measured separately using two different versions of the SC-IAT. Five words from the category *self* (e.g., ‘I’, ‘me’) were either paired with pictures representing the categories *compassion* and *neutral* (eSC-IAT) or *legal* and *illegal* (aSC-IAT). These category labels were used as according to Hare (2003), highly psychopathic individuals can be distinguished from others by their reaction to empathy-evoking stimuli (neutral emotional response instead of empathy) and their proneness to illegal activities (represented by different PCL-items, such as juvenile delinquency or criminal versatility). Twenty pictures (5 per category) were taken from the International Affective Picture System (IAPS; Lang et al., 2008). Self-describing words were either paired with stimuli from the category compassion (eSC-IAT) or legal (aSC-IAT) in the *compatible block*, whereas the same words were paired with stimuli from the categories neutral (eSC-IAT) or illegal (aSC-IAT) in the *incompatible block*. By consensus between two of the authors, 20 pictures were selected that most adequately represented the categories. In the eSC-IAT, empathy-evoking pictures (e.g., weeping children, injured animals) were contrasted with neutral pictures (e.g., a cup, clothespins), whereas in the aSC-IAT, depictions of either delinquent actions (such as theft or physical violence) or affectionate social interactions (e.g., conversations and hugs) were presented (see Supplementary material for a full list of all stimuli).

At the beginning, participants were informed that they were now completing a task that required them to sort stimuli into different categories. Both SC-IATs consisted of 2 stages with 24 practice trials and 72 test trials each. Answers were given by pressing either the *e* key or the *i* key, with left-and right-handed responses being approximately counterbalanced (i.e., 58% left-handed responses in the compatible block, 58% right-handed responses in the incompatible block). Category reminders were positioned on the top quarter of both sides of the screen. The stimuli appeared centered on the screen, in randomized order, and remained on screen for 3,000 ms. In case reaction times exceeded 1,000 ms, an instruction to respond faster was presented (“please respond faster!”). Furthermore, feedback signals indicated the accuracy of responses (i.e., green O for correct, red X for incorrect responses). Both the aSC-IAT (*r*_tt_ = 0.79) and the eSC-IAT (*r*_tt_ = 0.87) showed a sufficient split-half reliability (i.e., odd-even method) in this study.

Self-reported psychopathic traits were measured with the German translation of the SRP 4 (Mokros et al., 2016) in which psychopathy is captured through 64 items that are answered on a 5-point rating scale ranging from *strong disagreement* (1) to *strong agreement* (5). Apart from a total score, four subscale scores can be calculated that correspond to the four PCL-R facets: Interpersonal, Affective, Lifestyle, and Antisocial. Paulhus et al. (2016) reported high internal consistency coefficients of Cronbach’s α = 0.89 (community; *N* = 638) and 0.92 (college; *N* = 788). Test–retest correlations over a period of 10 weeks were of similar size (*r*_tt_ = 0.82; *n* = 48). In the present study, internal consistency coefficients for the SRP 4 total score and subscale scores (Cronbach’s α ≥ 0.78) were satisfactory, except for the Antisocial Scale (Cronbach’s α = 0.56).

#### Procedure and data analysis

Participants gave their informed consent and could either receive a course credit for their participation or take part in a raffle in which four individuals won a voucher of € 15 each. The study was conducted online using the survey package *SoSci Survey 2.0* (Leiner, 2019). To test for potential effects of experimental order, half of the sample completed the eSC-IAT first before providing demographic information and completing the aSC-IAT as well as the SRP 4. The order of the SC-IATs was exchanged for the other half of the participants. A debriefing about the rationale of the indirect measures and the aims of the study was administered in both conditions.

Data analysis for all four studies was conducted with IBM SPSS 26. *D-scores* were calculated for both SC-IATs, based on the standardized RT-difference between the test trials in blocks 2 and 4 only (*cf.* Karpinski and Steinman, 2006). Therefore, negative *D*-scores indicate an implicit self-concept of being more antisocial and less empathic, respectively. Data reduction was performed for the SC-IATs according to the recommendations of Karpinski and Steinman (2006; i.e., invalidation of cases with error rates above 20%; deletion of RTs below 350 ms and non-responses; use of a RT-penalty of 400 ms for erroneous trials). Furthermore, data sets with zero intraindividual variance in the SRP 4 (i.e., *SD* = 0) were removed to control for response bias (see Supplementary material for a more detailed description of preliminary and additional analyses). Associations between the SRP 4 and the SC-IATs were examined through Pearson product–moment correlational analyses. Except for H1 that included a logical disjunction (i.e., hypothesis is supported if at least one correlation is significant), all hypotheses tested in this study were specific and *a priori* hypotheses. In keeping with the guidelines proposed by Weber (2007), Bonferroni-corrected alpha-error-probabilities were thus calculated for H1 only (i.e., α = 0.05/2 = 0.025).

### Results

Correlations between the SRP 4 and the SC-IATs were exclusively negative. As expected, significant negative correlations between the eSC-IAT and the SRP 4 total score (*r* = −0.17; *p* = 0.005, 95% CI [−0.30, −0.04]) as well as the SRP 4 Interpersonal scale (*r* = −0.14, *p* = 0.018, 95% CI [−0.25, −0.02]) and the SRP 4 Affective scale (*r* = −0.16, *p* = 0.007, 95% CI [−0.29, −0.04]) were found. The aSC-IAT, in contrast, was neither correlated with the SRP 4 total score nor with any of the subscale scores (*ps* ≥ 0.05; see Table 1 for all correlation coefficients).

**Table 1:** Intercorrelations between all variables addressed in the hypotheses of Study 1.

	*M*	*SD*	α/*r*_tt_	2	3	4	5	6	7
1. aSC-IAT	0.22	0.36	0.79	0.05	−0.11	−0.08	−0.09	−0.08	−0.10
2. eSC-IAT	−0.05	0.42	0.86		−0.17 **	−0.14 *	−0.16 **	−0.12 *	−0.09
3. SRP 4 total	125.18	23.63	0.90			0.86 **	0.79 **	0.81 **	0.54 **
4. SRP 4 INT	36.33	9.38	0.84				0.66 **	0.52 **	0.30 **
5. SRP 4 AFF	31.31	7.53	0.78					0.48 **	0.20 **
6. SRP 4 LIF	36.39	8.50	0.78						0.42 **
7. SRP 4 ANT	21.15	5.06	0.56						

*N* = 227. aSC-IAT, Antisociality Single Category Implicit Association Test; eSC-IAT, Empathy Single-Category Implicit Association Test; SRP 4, Self-Report Psychopathy Scale; Total, total score; INT, Interpersonal; AFF, Affective; LIF, Lifestyle; ANT, Antisocial. **p < 0.01 (one-sided); *p < 0.05 (one-sided).

### Discussion

The purpose of Study 1 was to explore the relationship between psychopathy and the implicit self-concept in an online sample. Therefore, two separate measures of the implicit self-concept regarding empathy and antisociality were developed that showed split-half reliabilities comparable to the average reliability previously observed for self-concept IAT-measures (*r*_tt_ = 0.70, *k* = 44; De Cuyper et al., 2017). The SC-IAT results – weaker associations of the self with compassion than neutral – were weakly but significantly correlated to the SRP 4 total, Interpersonal and Affective scores. In contrast, neither the SRP 4 total, nor the Lifestyle or Antisocial scores were related to stronger associations between the self and illegal than legal. The results were thus in accordance with H1.1 and H1.2 (negative correlation between eSC-IAT and SRP 4 Interpersonal and Affective scale), whereas H1.3 and H1.4 (negative correlation between aSC-IAT and SRP 4 Lifestyle and Antisociality scale) were refuted. H1 (negative correlation between SC-IATs and SRP4 total score) could be upheld for the eSC-IAT only.

Although being comparable to the average explicit-implicit correlations for SC-IATs (*r* = 0.17; *k* = 6; De Cuyper et al., 2017), the relationships observed in the present study were weak, with effect sizes (*r*) ranging from 0.10 to 0.20. This rather small magnitude could have resulted from sample characteristics: A lower average SRP 4 score compared to prior studies (Paulhus et al., 2016; *d* = 0.57) suggests a potential floor effect, possibly due to the large percentage of females in the present sample (*d* = 0.64 for sex effects on the SRP 4 total score). In addition, the study design did not allow for in-depth explanations regarding the nature of SC-IAT effects. As prior studies found a relationship between psychopathy and impaired response reversal (Budhani et al., 2006), SC-IAT effects could have resulted from an incapacity to switch reward-contingencies between the experimental blocks. Furthermore, rather than representing a deviant self-concept, our findings might simply reflect emotion processing deficits (for a review, see Brook et al., 2013) that could have hindered highly psychopathic participants from accurately categorizing the pictures. To control for these possible methodological artifacts, we replicated Study 1 in an independent sample with corresponding changes in the study design.

## Study 2

Study 2 was designed as a conceptual replication of Study 1, in which a more heterogeneous distribution of psychopathic traits was sought through increasing the number of male subjects. Furthermore, two simple picture categorization tasks (CTs) were implemented in order to control for the putative effects of any difficulty in picture recognition or task-switching. Moreover, the temporal stability of the implicit self-concept was tested by asking the participants to complete both SC-IATs and the SRP 4 twice, with a retest interval of 6 weeks. Positive correlations between the SC-IATs at both measurement time points were expected (H1.1 and H1.2). In addition, the SC-IATs were expected to be incremental in the prediction of the SRP 4 total scores above and beyond the CTs: The eSC-IAT was expected to be incremental above and beyond a compassion vs. neutral CT (H2.1), whereas the aSC-IAT was expected to be incremental above and beyond a legal vs. illegal CT (H2.2).

Convergent validity of the implicit self-concept was assessed in terms of differential correlations with external criteria. These criteria included the traits described in the HEXACO model of personality (Ashton and Lee, 2009). Both the Affective and the Antisocial facets of the PCL-R have been found to be negatively correlated with the HEXACO personality traits Honesty-Humility, Emotionality, Agreeableness, and Conscientiousness in previous studies, whereas Extraversion has been (negatively) related to the Affective facet (Međedović and Petrović, 2015; Garofalo et al., 2019).

Therefore, positive relationships between the eSC-IAT and Emotionality, Extraversion, and Honesty-Humility were expected (H3.1, H3.2 and H3.3). For the aSC-IAT, significant correlations with Conscientiousness (H4.1), Agreeableness (H4.2), and Honesty-Humility (H4.3) were assumed. As both antisociality and impulsivity have been linked to the PCL-R Antisocial facet in prior research (e.g., Coid et al., 2009; Hollerbach et al., 2020), significant correlations were expected between the aSC-IAT and both impulsivity (H5.1 to H5.4) and antisocial behavior (H5.5). Given the non-significant correlations between the aSC-IAT and the SRP 4 in Study 1, no directional hypotheses were formulated for the aSC-IAT^4^.

### Materials and methods

#### Participants

Based on the average test–retest correlation in previous studies (*r_tt_* = 0.26, *k* = 5; own calculation described in the Supplementary material) and the average IAT-explicit correlation (*r* = 0.21, *k* = 155; Greenwald et al., 2009), a small effect size (*r* = 0.21) was expected prior to the experiment that would require a minimum sample size of 139 individuals according to G*Power 3.1.9.2 (actual power of 80% type I error probability of 5%). The attrition-adjusted target sample size was 360–420 participants for measurement time point 1 (*t*_1_), and 139 participants for measurement time point 2 (*t*_2_). Owing to technical problems, sample sizes were restricted to 230 participants at *t*_1_ (59% female) and 112 participants at *t*_2_ (64% female), affording an effective power (with the parameters mentioned above) of 95% (*t*_1_) and 73% (*t*_2_), respectively. After exclusions, the final samples consisted of 219 participants at *t*_1_ (60% female, *M*_age_ = 40.36, *SD*_age_ = 12.74) and 110 participants at t_2_ (66% female, *M*_age_ = 41.37, *SD*_age_ = 12.62; see Supplementary material).

#### Instruments

The same SC-IATs as implemented in Study 1 were used, with minor revisions. In line with previous studies (e.g., Chevance et al., 2017; Nentjes et al., 2017), another 10 practice trials were added at the beginning of each SC-IAT, which were not analyzed (202 trials overall). The response time-out and the instruction to respond faster were deleted as potential task-switching costs might further increase under time-restriction. The stimuli were presented in pseudo-randomized instead of randomized order. Further, extended versions of the single categorization blocks were implemented to control for methodological artifacts. These categorization tasks consisted of 100 trials each, in which 100 different pictures (50 per category) were assigned to the categories compassion and neutral (*empathy categorization task*, eCT) or legal and illegal (*antisociality categorization task*, aCT) within a response-frame of 1,500 ms. The pictures were taken from the IAPS (including the stimuli used in the SC-IATs) as well as a free picture database. As reward-contingencies did not change within this task (i.e., no combination of categories, no change from compatible to incompatible trials), task-switching costs should be minimal. A feedback was given when reaction times exceeded 1,500 ms (“too slow”), whereas no feedback was given regarding the accuracy of responses. Split-half reliabilities at both measurement occasions for the eSC-IAT (*r*_tt_ = 0.80 and *r*_tt_ = 0.84) and the aSC-IAT (*r*_tt_ = 0.78 and *r*_tt_ = 0.77) were again sufficient, whereas coefficients for the eCT (*r*_tt_ = 0.74) and the aCT (*r*_tt_ = 0.66) were slightly lower.

Psychopathy was again measured through the SRP 4 (*t*_1_: Cronbach’s *α* = 0.59–0.87; *t*_2_: Cronbach’s *α* = 0.54–0.86). General personality traits were assessed using the HEXACO-60 (Ashton and Lee, 2009), a self-report questionnaire in which each of the six HEXACO traits (Honesty-Humility, Emotional Stability, Extraversion, Agreeableness, Conscientiousness, and Openness) is captured by 10 items, answered on a 5-point rating scale ranging from 1 = *strong disagreement* to 5 = *strong agreement*. Impulsivity was assessed through the 45 items of the Brief UPPS impulsive behavior scales (Whiteside and Lynam, 2001; German version by Keye et al., 2009) which is named after its four subscales: Urgency, (lack of) Premeditation, (lack of) Perseverance, and Sensation Seeking. In contrast to the original version, the coding of certain items was changed so that higher UPPS scores indicate higher levels of impulsivity (i.e., high urgency, low premeditation, low perseverance, and high sensation seeking).

Previous studies have demonstrated good reliability and factorial validity for the use of German versions of the HEXACO-60 (Moshagen et al., 2014) and the UPPS scales (Keye et al., 2009) in community samples. Aligning with these results, satisfactory reliabilities for the HEXACO (Cronbach’s *α* ≥ 0.68) and the UPPS subscales (Cronbach’s *α* ≥ 0.81) were observed in the present study. Antisocial propensity was captured via the Antisocial Behavior Questionnaire (ANTIQUE), a newly developed measure by Allen et al. (in preparation). In the ANTIQUE, the frequency of 14 different types of antisocial behavior (e.g., threatening or humiliating someone, shoplifting, sexual assault) within the last 3 years is reported on a 4-point scale (1 = *1–2 times*, 2 = *3–5 times*, 3 = *6–10 times*, 4 = *more than 10 times*). Although not established in prior research, its reliability was suggested by satisfactory coefficients in this study (Cronbach’s *α* = 0.70).

#### Procedure and data analyses

Both measurement time points were completed online using the software packages *EFS Survey, Version Summer 2017* (EFS Survey, 2017) and *Inquisit 5* (Inquisit, 2016). Participants were informed about the content, the duration, the conditions of participation (see preregistration form for all exclusion and inclusion criteria), and the follow-up design using a personalized code list with e-mail addresses. The experiment started with the SC-IATs and the CTs in Inquisit, followed by the questionnaires as well as demographic questions in EFS Survey. Participants were again either rewarded with a course credit or the possibility to take part in a raffle for three vouchers. Exactly 6 weeks later, the participants were contacted via e-mail and asked to complete the second part of the experiment within 1 week, consisting of both SC-IATs in Inquisit and the SRP 4 in EFS Survey. All participants were fully debriefed at the end of *t*_2_ session.

Before the analyses, data sets collected at *t*_1_ and *t*_2_ were combined using the personal codes generated at the end of each measurement. Missing values in the questionnaires were replaced by the individual mean on the respective subscale (i.e., mean imputation) and data sets with missing values in more than 5% of the items were excluded. Data sets were excluded in case of self-reported non-serious participation, zero intraindividual variance in any of the questionnaires except the ANTIQUE, or an error-rate above 20% in either of the SC-IATs (for further details see Supplementary material). The SC-IATs were again analyzed in terms of *D*-scores, with RTs above 3,000 ms being excluded for reasons of comparability with Study 1. For the CTs, *D*-score equivalents following a similar metric were computed (i.e., RTs below 350 and above 1,500 ms discarded). H1, H3, H4, and H5 were tested via correlation analyses, with one-tailed testing was used for directional and two-tailed testing was for non-directional hypotheses. H2 (incremental validity of the SC-IATs) was tested using hierarchical linear regression (method: Enter). Separate regression equations were calculated with either aCT entered in step 1 and aSC-IAT in step 2 (H2.1) or eCT entered in step 1 and eSC-IAT entered in step 2 (H2.2). As in Study 1, no type I-error correction was carried out (see Supplementary material for further details).

### Results

As positive test-retest correlation coefficients were observed for the eSC-IAT (*r*_tt_ = 0.40, *p* < 0.001, 95% CI [0.26, 1]) and the aSC-IAT (*r*_tt_ = 0.31, *p* < 0.001, 95% CI [0.16, 1]), both hypotheses on the stability of the implicit self-concept (H1.1 and H1.2) were supported. In contrast, mostly non-significant correlations were observed between the SC-IATs and the self-report measures (see Supplementary Table S1). Therefore, the hypotheses on convergent validity with impulsivity and general personality traits (H3, H4 H5 and all sub-hypotheses) had to be rejected.

H2.1 and H2.2 were tested through regression analyses. Prior to these analyses, correlations between the CTs, both SC-IATs, and the SRP 4 were calculated. A single significant correlation was observed between the eSC-IAT and the eCT (*r* = 0.14, *p* = 0.034, 95% CI [0.01, 0.27]). Consequently, neither the aCT (adjusted *R*^2^ = −0.00) nor the eCT (adjusted *R*^2^ = 0.01) significantly contributed to explaining variance of the SRP 4 total scores when entered at step 1. Entering the SC-IAT scores at step 2 resulted in no significant increase in *R*^2^ (Δ*R*^2^ = 0.00 for both regression analyses; see Supplementary material for further details, including a more sophisticated analysis of temporal stability). H2.1 and H2.2 were thus not supported.

### Discussion

Study 2 was designed as a replication of Study 1 in a more heterogeneous sample using additional validation criteria and a follow-up design. Variance in psychopathy scores should be increased by increasing the proportion of male participants (41% at *t*_1_ and 36% at *t*_2_, as compared to 21% in Study 1). Nevertheless, the SC-IATs were unrelated to the SRP 4 total score and all other variables, implying that neither psychopathy nor its external correlates were related to a self-concept of being more antisocial and less empathic. Accordingly, neither H3, nor H4, nor H5 (positive correlations between the eSC-IAT and Emotionality, Extraversion, and Honesty-Humility; significant correlations between the aSC-IAT and Conscientiousness, Agreeableness, Honesty-Humility, impulsivity, and antisocial behavior) could be upheld.

To control for effects of picture recognition or task-switching deficits, the SC-IATs were compared to two less complex CTs. Contrary to our hypotheses, incremental validity of the SC-IATs could not be established. H2.1 and H2.2 (incremental validity of the SC-IATs in the prediction of the SRP 4 total scores above and beyond the CTs) had to be refuted and it still remains unclear whether the significant correlations observed in Study 1 in fact resulted from methodological artifacts. In contrast, temporal stability as assumed in H1.1 and H1.2 (positive correlations between the SC-IATs at both measurement time points) was established for the implicit self-concept. Test–retest correlations over an interval of 6 weeks were relatively weak, but higher than the average stability of SC-IATs in previous studies (see also Greenwald and Lai, 2020). This finding was corroborated by the results of a more complex analysis of stability (see Supplementary material).

Despite several improvements, some methodological constraints remained in Study 2, with the most striking being the relatively low SRP 4 total scores compared to previous research (*d* = 0.49; Paulhus et al., 2016; college sample). In addition, the predetermined minimum sample size of 139 participants was not achieved at both measurement time points. Although *post hoc* power analyses implied a moderate to satisfactory statistical power of 73% for detecting a test–retest correlation of *r*_tt_ = 0.21 (see H1.1. and H1.2), the multivariate analyses (i.e., test of incremental validity) would have required a larger sample size. Thus, a field study was conducted with samples of offenders and community participants.

## Study **3**

Study 3 comprised two distinct replications of Study 1 (i.e., Study 3a) and Study 2 (i.e., Study 3b). As antisociality is obviously more prevalent in samples of incarcerated offenders (Blais et al., 2014) and presumably also in practitioners of power sports (e.g., Endresen and Olweus, 2005; Breitschuh et al., 2018), Study 3 aimed at further increasing sample heterogeneity by recruiting a mixed sample of offenders as well as community participants with and without experience in power sports. The assessment of psychopathy, in addition, was extended with observer-report measures.

### Hypotheses Study 3a

Based on the results of Study 1, the eSC-IAT was expected to be negatively correlated with the PCL-R/PCL:SV total score (H1.1) as well as the Interpersonal (H1.2) and the Affective (H1.3) facets. The aSC-IAT, in contrast, was assumed to be negatively correlated with the PCL-R/PCL:SV total score (H1.4), the Lifestyle (H1.5) and the Antisocial facets (H1.6). The PCL-R/PCL:SV total and facet scores were expected to be positively correlated with the corresponding scores of the SRP 4 (H2.1 to H2.5). Moreover, it was assumed that incorporating the implicit self-concept in psychopathy assessment would be beneficial, resulting in incremental validity of a combination of the SC-IATs and the SRP 4 in the prediction of the PCL-R/PCL:SV total and facet scores (H3) above and beyond the SRP 4 alone.

It was further tested whether subgroups of participants could be distinguished based on their SC-IAT scores. In latent class analyses of the PCL-R, it was repeatedly possible to discriminate between individuals with high scores on all facets and individuals with either high scores on the Interpersonal and Affective facets, or high scores on the Lifestyle and Antisocial facets only, or with low scores on all facets (Krstic et al., 2018; Lehmann et al., 2019). Therefore, lower eSC-IAT scores were expected in participants with primarily affective (H4.1) and both affective and antisocial deficits (H4.3) compared to those with primarily antisocial deficits or without deficits. Regarding the aSC-IAT, lower scores were expected for participants with antisocial (H4.2) and both deficits (H4.4) compared to those with only affective or no deficits. As recent studies suggest relationships between psychopathy and response inhibition deficits (Weidacker et al., 2017) and between general mental ability and emotion perception deficits among highly psychopathic participants (Olderbak et al., 2018), both variables served as covariates.

### Hypotheses Study 3b

A subsample of the participants tested in Study 3a was re-assessed approximately 1 year later with the same measures used in Study 2. Temporal stability of the eSC-IAT (H1.1) and the aSC-IAT (H1.2) was assumed in terms of positive test–retest correlations. Furthermore, it was again hypothesized that the eSC-IAT would be positively correlated with Honesty-Humility, Emotionality, and Extraversion (H3.1 to H3.3), whereas the aSC-IAT should be significantly correlated with Agreeableness, Conscientiousness, Honesty-Humility, self-reported impulsivity, and antisociality (H4.1 to H5.5). In addition, significant relationships between the aSC-IAT and the overall number and versatility of misconduct in the correctional institutions were expected (H5.6 and H5.7; *cf.* Olver et al., 2020). Regarding the prediction of PCL-R total scores, the eSC-IAT was assumed to be incremental compared to the eCT (H2.1) and the aSC-IAT to be incremental compared to the aCT (H2.2).

### Materials and methods

#### Participants Study 3a

G*Power 3.1.9.2 (Faul et al., 2009) suggested a minimum sample size of 150 to 207 required to ensure a type I error rate of 5% and a statistical power of 80 to 90%, and to detect a small effect size (*r* = 0.20). In sum, 166 complete data sets (28% female, *M*_age_ = 36.27, *SD*_age_ = 10.61^5^) were collected over a period of approximately 20 months. The offender sample consisted of 28 male patients of a forensic-psychiatric hospital and 51 male inmates recruited in four prisons (*n* = 79; *M*_age_ = 37.29, *SD*_age_ = 10.71)^6^. Data collection took place in Germany. The community subsample comprised 87 participants (54% female, *M*_age_ = 35.49, *SD*_age_ = 10.53) of whom 23% were active in martial arts (e.g., Mixed Martial Arts, Karate, Krav Maga). The final sample size after data reduction was 162 (47% offenders, 29% females, *M*_age_ = 36.16, *SD*_age_ = 10.67; see Supplementary material for further details).

#### Participants Study 3b

As attrition effects were likely given the long test–retest interval, we sought to achieve a sample size between 40 and 48 participants for Study 3b. In sum, 47 participants of the offender sample (*M*_age_ = 39.62, *SD*_age_ = 12.12) were recruited in four of the five facilities (3 prisons) and tested approximately 1 year after Study 3a (*M* = 12.21 months, *SD* = 1.67, range = 9 to 14 months). The final sample sizes after data preprocessing and exclusions were 39 (*M*_age_ = 39.62, *SD*_age_ = 12.83) for the analysis of H1 and H3, and 38 (*M*_age_ = 39.39, *SD*_age_ = 12.93) for the analysis of H2 (see Supplementary material for further details).

#### Instruments Study 3a

PCL-R/PCL:SV interviews were conducted by nine trained raters, seven of whom administered the PCL-R and four the PCL:SV interviews. According to Hare et al. (2013), equivalence between both measures allows for transformations of PCL:SV scores to PCL-R scores and vice versa. Collateral file information was only available for the rating of the PCL-R in the offender subsample. Participants were rated on 20 items using a 3-point-scale between 0 and 2. The items were aggregated into a total score and into four facets and two factor scores. Community participants were rated on the 12 items of the PCL:SV, a variant of the PCL-R that allows for assessing psychopathy in both offender and community samples (for examples see Weidacker et al., 2017; Olderbak et al., 2018). For both measures, interrater reliability was estimated using the one-way random model (ICC_1,1_)^7^. Agreement was substantial to strong for the PCL-R total (0.94), and facet scores (0.69–0.92, *n* = 36), the PCL:SV total score (0.89), and all PCL:SV facets (0.83–1.00) except the Lifestyle facet (0.39, *n* = 13).

The self-report assessment of psychopathy was again based on the German version of the SRP 4 that had been found sufficiently reliable (Cronbach’s α = 0.64–0.89) and strongly correlated with the PCL-R (*r* ≥ 0.62) in an offender sample (Hollerbach et al., 2020; *N* = 117). Reliability in the present sample was high for the total score and the subscales (Cronbach’s α = 0.82–0.94). General mental ability was measured with the *Wiener Matrizen Test 2* (WMT-2; Formann et al., 2011), in which participants are asked to complete matrices of geometrical symbols. The WMT-2 is a power test, with different item difficulties and no time limit. The 18 items of the WMT-2 were aggregated to a sum score. According to Formann et al. (2011), high internal consistency (Cronbach’s α = 0.82) was observed in the validation sample (*N* = 2,494). Hundred and fifty participants completed the WMT-2. Hundred and twenty-seven WMT-2 data sets (42% offenders) were analyzed after excluding participants with missing data. Internal consistency of the WMT-2 was high in the present sample (Cronbach’s α = 0.83).

Both relevant parts of the implicit self-concept were measured through lab versions of the SC-IATs used in Study 2 (response keys *y* and *– [dash]*). A third SC-IAT (*control SC-IAT*; cSC-IAT) was implemented to control for effects of response inhibition deficits. For the cSC-IAT, IAPS-pictures of flowers were paired with 10 either positive (e.g., ‘happy,’ ‘pleasure,’ ‘wonderful’) or negative words (e.g., ‘nasty,’ ‘evil,’ ‘disgusting’) adapted from Karpinski and Steinman (2006). Pictures of flowers were paired with good words in the compatible block and with bad words in the incompatible block. Split-half reliability coefficients were satisfactory to high for the aSC-IAT (*r*_tt_ = 0.74) the cSC-IAT (*r*_tt_ = 0.75), and the eSC-IAT (*r*_tt_ = 0.90).

#### Instruments Study 3b

Unaltered versions of the eSC-IAT (*r*_tt_ = 0.85) and the aSC-IAT (*r*_tt_ = 0.83) were implemented along with the eCT (*r*_tt_ = 0.79), the aCT (*r*_tt_ = 0.60), and the questionnaires (Cronbach’s α ≥ 0.73) used in Study 2. The overall number and versatility of instances of institutional misconduct were recorded from files. Seventeen different types of misconduct were coded and aggregated to five broad categories following the concept of offense-analogue behavior by Gordon and Wong (2015): Interpersonal aggression, substance abuse, work ethic, security level, and emotional control. On a higher level, these categories were ascribed to three superordinate scales labeled as violent misconduct (interpersonal aggression), substance related misconducts (substance abuse), and non-violent misconducts (work ethic, security level, and emotional control).

#### Procedure and data analysis Study 3a

The consent forms again included information on the purpose, the content, the approximate duration, the inclusion and exclusion criteria (for all criteria, see preregistration form), and the coding list that was used in order to combine scores on the different instruments (i.e., self-and observer-reports, indirect measures) as well as from both measurement occasions (Study 3a and 3b). The experiment consisted of two separate blocks, with one block including the PCL-R/PCL:SV interview and the other including self-report and indirect measures administered on a computer using *Inquisit* 5. Both blocks were either completed subsequently with a short break in between or at two different occasions. The block order could not be held constant.

The computer block had a fixed order starting with the eSC-IAT, the aSC-IAT, and the cSC-IAT. In the community sample, the SC-IATs were followed by the WMT-2, and the SRP 4. In the offender sample, WMT-2 scores were partly adopted from a related project (Hauser et al., 2021) and the SC-IATs were followed by the SRP 4. Demographic information was collected and a debriefing was offered to all participants at the end of the experiment. Participants were either financially compensated depending on the duration of the experiment (15€ at maximum) or with a course credit.

PCL:SV scores were transformed to PCL-R scores through proportional multiplication with the factor 1.67 (total score, Factor 1, Lifestyle, and Antisocial) or 1.33 (Factor 2, Interpersonal and Affective), respectively. Hypothesis testing was performed through bivariate correlation analyses (H1 and H2) and hierarchical regression analyses, in which the relevant SRP 4 scores were entered first, with the aSC-IAT and eSC-IAT scores being added in the second step (H3). Alpha-error adjustments were performed for H3 only as this was the only hypothesis including a logical disjunction (i.e., 0.05/5 = 0.01). Given the relatively low percentage of elevated scores in the overall sample, subsample sizes for the groups addressed in H4 (solely affective or antisocial deficits, deficits in both areas, or no salient deficits) differed strongly. The groups were therefore collapsed and two independent samples *t*-tests were calculated to compare (a) participants with elevated scores on PCL-Factor 1 (*n* = 46) and all other participants (*n* = 116) regarding their scores on the eSC-IAT and (b) participants with elevated scores on PCL-Factor 2 (*n* = 44) and all other participants (*n* = 118) regarding their scores on the aSC-IAT. In other words, H4.1 and H4.3 as well as H4.2 and H4.4 were tested simultaneously (see Supplementary material for further details).

#### Procedure and data analysis Study 3b

Fifty-four participants of Study 3a sample were contacted again, of whom 47 (87%) gave their informed consent to participate in Study 3b. All materials were presented electronically using *Inquisit 5*. The first block of the experiment included the eSC-IAT, the aSC-IAT, the eCT, and the aCT. If not already available from Study 3a, WMT-2 data was collected subsequently, and all participants completed the questionnaires. Demographic questions were answered at the end of the experiment, followed by the debriefing. Participants were compensated with 15€. Hypotheses testing was again based on bivariate correlations (H1, H3, H4, and H5) and hierarchical linear regression analyses (H2.1 and H2.2). We again abstained from correcting for type I-error cumulation (see Supplementary material for further details).

### Results

#### Results of Study 3a

As presented in Table 2, the eSC-IAT was unrelated to the PCL-R total, Interpersonal, and Affective facet scores. Correlations between the aSC-IAT score and both the PCL-R total and Antisocial facet scores were not significant, either (all *ps* > 0.05). Unexpectedly, the PCL-R Lifestyle facet score showed a significant positive correlation with the aSC-IAT score (*r* = 0.15, *p* = 0.033, 95% CI [−0.00, 0.30]). H1.1 to H1.6 were thus rejected. In line with H2.1 to H2.5, all PCL-R scores were positively correlated with the respective SRP 4 scores (all *r* coefficients ≥0.39, all *p*s = 0.000).

**Table 2:** Zero-order correlations between all variables addressed in the hypotheses of Study 3a.

	*M*	*SD*	α/*r*_tt_	2	3	4	5	6	7	8	9	10	11	12
1. eSC-IAT	0.05	0.39	0.90	0.16^‡^*	−0.09^‡^	−0.09^‡^	−0.07^‡^	−0.12^‡^	−0.09 ^‡^	−0.02 ^‡^	−0.04 ^‡^	−0.06 ^‡^	0.06 ^‡^	−0.03 ^‡^
2. aSC-IAT	0.03	0.31	0.74		0.10 ^‡^	0.15^‡^*	0.09 ^‡^	0.15^‡^*	0.06 ^‡^	0.00 ^‡^	−0.01 ^‡^	0.00 ^‡^	0.01 ^‡^	0.01 ^‡^
3. PCL-R total	12.05	11.36	–			0.77^a^**	0.91^†^**	0.83^†^**	0.82^†^**	0.72^†^**	0.36^†^**	0.54^†^**	0.51^†^**	0.81^†^**
4. PCL-R INT	2.45	2.38	–				0.69^†^**	0.54^†^**	0.48^†^**	0.50^†^**	0.39^†^**	0.42^†^**	0.35^†^**	0.45^†^**
5. PCL-R AFF	2.80	3.01	–					0.71^†^**	0.70^†^**	0.57^†^**	0.25^†^**	0.44^†^**	0.35^†^**	0.71^†^**
6. PCL-R LIF	3.08	3.22	–						0.82^†^**	0.62^†^**	0.24^†^**	0.43^†^**	0.49^†^**	0.74^†^**
7. PCL-R ANT	2.65	3.36	–							0.70^†^**	0.29^†^**	0.47^†^**	0.53^†^**	0.83^†^**
8. SRP 4 total	147.74	35.49	0.94								0.75^†^**	0.82^†^**	0.87^†^**	0.82^†^**
9. SRP 4 INT	38.12	9.29	0.84									0.60^†^**	0.65^†^**	0.35^†^**
10. SRP 4 AFF	35.77	9.02	0.82										0.63 ^†^**	0.53^†^**
11. SRP 4 LIF	42.12	10.51	0.83											0.59^†^**
12. SRP 4 ANT	31.73	14.69	0.90											

eSC-IAT, empathy Single-Category Implicit Association Test; aSC-IAT, antisociality Single-Category Implicit Association Test; PCL-R, Psychopathy Checklist-Revised; SRP 4, Self-Report Psychopathy Scale; Total, total score; INT, Interpersonal; AFF, Affective; LIF, Lifestyle; ANT, Antisocial. †*n* = 166. ^‡‡^*n* = 162. **p < 0.01; *p < 0.05.

Regarding H3, a combination of the SRP 4 Lifestyle score and both SC-IATs was incremental in the prediction of the PCL-R Lifestyle facet score above and beyond the corresponding SRP 4 facet score (Δ*R*^2^ = 0.05, *p* = 0.005). Only the SRP 4 Lifestyle facet score exceeded the predetermined significance level in step 2, whereas neither of the two SC-IAT scores were significant predictors in the model. Regarding all other PCL-R scores, a combination of SRP 4 and SC-IATs was not superior to the SRP 4 alone (all *p*s > 0.05; see Supplementary material). H3 was thus only supported regarding the PCL-R Lifestyle facet.

Independent samples *t*-tests indicated that average eSC-IAT scores were not significantly lower in participants with elevated PCL-R Factor 1 scores compared to all other participants (*t*[160] = 0.04, *p* = 0.485, *d* = 0.01, 95% CI [−0.33, 0.35]). Similarly, average aSC-IAT scores were not significantly lower among participants with elevated PCL-R Factor 2 scores compared to all other participants (*t*[160] = −1.50, *p* = 0.069, *d* = −0.26, 95% CI [−0.61, 0.09]). H4 was thus not supported. Additional analyses showed that these effects were stronger in the offender sample, and when WMT-2 scores were considered as a covariate (see Supplementary material for further details).

#### Results of Study 3b

Contrary to our expectations, test–retest correlations of the eSC-IAT (*r*_tt_ = 0.22, *p* = 0.093, 95% CI [−0.10, 0.50]) as well as the aSC-IAT (*r*_tt_ = 0.12, *p* = 0.232, 95% CI [−0.20, 0.42]) were not statistically significant. Furthermore, convergent validity could not be ascertained as the eSC-IAT score was not significantly correlated with HEXACO Emotionality, Extraversion, or Honesty-Humility, just as the aSC-IAT was not significantly correlated with HEXACO Conscientiousness, Agreeableness, and Honesty-Humility, the UPPS scales, the ANTIQUE, or the overall number or versatility of rule violations (all *p*s > 0.05). Neither could incremental validity be demonstrated. Unexpectedly, the eCT score alone (step 1), as well as a combination of the eCT score and the eSC-IAT score (step 2) did not explain a substantial amount of variance in the PCL-R score measured at *t*_1_. The same was true for the aCT score (step 1) and a combination of the aCT and the aSC-IAT score (step 2, all *p*s > 0.05; see Supplementary material for a more detailed description and all preliminary and additional analyses). Therefore, all hypotheses had to be rejected.

### Discussion

Study 3a also failed to replicate the results of Study 1. In a sample consisting of offenders and community participants, relationships between the eSC-IAT and all PCL-R scores were negative, but non-significant (*p*s > 0.05). The aSC-IAT, in contrast, was unrelated to the PCL-R total score and the PCL-R Antisocial facet score, but positively correlated with the PCL-R Lifestyle facet score. Hence, H1.1 to H1.6 (negative correlations between eSC-IAT and PCL-R/PCL:SV total score, Interpersonal and Affective facets; negative correlations between aSC-IAT and PCL-R/PCL:SV total score, Lifestyle and Antisocial facets) had to be rejected. All SRP 4 scores were, however, positively correlated with the respective scores on the PCL-R to a significant degree, thus supporting H2.1 to H2.5 (positive correlations between the PCL-R/PCL:SV total and facet scores and the corresponding scores of the SRP 4). When compared to the sole use of the SRP 4, a combination of both SC-IATs and the SRP 4 was nevertheless incremental in the prediction of the PCL-R Lifestyle facet score, thus supporting H3 (incremental validity of a combination of SC-IATs and SRP 4 in the prediction of PCL-R/PCL:SV total and facet scores above and beyond SRP 4 alone) at least partially. Although the effect size was small (Δ*R*^2^ = 0.05), our findings suggest that the implicit self-concept explains variance in psychopathy that is not attributable to the explicit self-concept alone. It was not possible, though, to distinguish participants with different patterns of predominant deficits based on their scores on the indirect measures (H4.1–4.4: Lower eSC-IAT scores in participants with primarily affective and both affective and antisocial deficits compared to those with primarily antisocial deficits or no deficits; lower aSC-IAT scores in participants with antisocial and both deficits compared to those with only affective or no deficits).

The most striking result of Study 3a was a weak positive relationship between psychopathy and the aSC-IAT score, suggesting that the implicit self-concept of highly psychopathic individuals does not reflect their documented proclivity for antisocial behavior. Highly psychopathic individuals have been described as being emotionally less concerned and feeling less responsibility for their prior criminal behavior (Hare, 2003). The aSC-IAT score might thus not be an indicator of antisocial propensity, but the extent to which actual antisocial behavior is internally attributed. This interpretation was further corroborated by the fact that the aSC-IAT was also correlated with the PCL-R Interpersonal and Affective facets within the offender sample.

Study 3b was a replication of Study 2, in which test–retest correlations of the SC-IATs were again unsatisfactorily low and the measures were not incremental in the prediction of scores on a psychopathy measure compared to the CTs. Even more critically, both SC-IAT scores were unrelated to constructs that had been linked to either the PCL-R Affective or Antisocial facet in the past, including the HEXACO personality traits, impulsivity, and antisociality. Therefore, all hypotheses (H1.1 and H1.2: positive test–retest correlations of both SC-IATs, H2.1 and H2.2: Incremental validity of eSC-IAT and aSC-IAT above and beyond eCT and aCT; H3.1 to H3.3: Positive correlations between eSC-IAT and Honesty-Humility, Emotionality, and Extraversion; H4.1 to H5.7: Significant correlations between aSC-IAT and Agreeableness, Conscientiousness, Honesty-Humility, self-reported impulsivity, antisociality, overall number and versatility of institutional misconduct) were rejected, raising serious concerns regarding the reliability and validity of the SC-IATs for the assessment of the implicit self-concept.

Still, these results might also be artifacts caused by limitations in the design of the studies, such as a variation in the experimental order (Study 3a) and an unsatisfactory sample size (Study 3b). Furthermore, we recommend replications with a shorter test–retest interval as with an increasing timespan, test–retest correlations generally tend to decrease while the risk of attrition effects and treatment-related changes in, for instance, trait self-control or executive functioning increases.

## General discussion

The studies described in this article were conducted to investigate the relationships between psychopathy and the implicit self-concept. Two adaptions of the SC-IAT were developed in order to capture the aspects of the implicit self-concept that are related to empathy and antisociality. Across all studies, the explicit self-concept regarding psychopathy was assessed using a questionnaire. The eSC-IAT was negatively correlated to this self-report measure in an initial validation study (Study 1), whereas both SC-IATs were relatively unstable, unrelated to psychopathy, impulsivity, and other personality traits in a cross-validation study (Study 2). In an experimental field study with incarcerated offenders and community controls (Study 3a), the eSC-IAT was unrelated to the PCL-R, but the aSC-IAT was positively correlated with the PCL-R Lifestyle facet. Moreover, a combination of the SC-IATs and the self-report was incremental in the prediction of the PCL-R Lifestyle facet compared to the self-report alone. These effects were more pronounced in the offender sample and when general mental ability was partialed out. When 47 participants were revisited approximately 1 year after Study 3a (Study 3b), again no evidence was found for test–retest reliability, convergent validity, or predictive power of the SC-IATs.

A striking explanation of these results is that the SC-IAT is not a viable measure of the implicit self-concept regarding psychopathy. This would also explain the unsatisfactory results observed in previous studies (Nentjes et al., 2017) and shift the focus on other indirect measures, such as the traditional IAT. However, other explanations also seem worth considering.

### Conceptual implications

The main purpose of this study was to explore the implicit self-concept of highly psychopathic individuals. Assuming that the SC-IATs were actually measures of the implicit self-concept, our results were counterintuitive. Based on the PCL-R model of psychopathy (Hare, 2003), we had expected negative relationships between both SC-IATs and psychopathy. The eSC-IAT scores were, however, negatively related to scores on the SRP 4 in one single study only (i.e., Study 1). The aSC-IAT scores, on the other hand, were also related to the PCL-R in only one study (i.e., Study 3a), but positively (i.e., contrary to expectation). Although being more antisocial at the behavioral level, highly psychopathic participants in Study 3a seemingly perceived themselves as being less antisocial than less psychopathic participants. This finding could be cautiously interpreted as indicating a self-concept of being rather less antisocial in highly psychopathic offenders, which would be at odds with prior studies, in which highly psychopathic individuals explicitly described themselves as rather more antisocial (e.g., Miller et al., 2014).

Our findings do align, however, with Suter et al. (2014, 2017) who also observed differential relationships between the implicit self-concept and psychopathy in different samples. Instead of antisociality, elevated aSC-IAT scores might actually indicate low levels of remorse, guilt, and responsibility (see Hare, 2003). Comparing themselves to other offenders, highly psychopathic offenders presumably protect their positive self-image by externalizing rather than internalizing the reasons for their prior convictions, thus viewing themselves as rather less antisocial. In prior studies, highly psychopathic offenders were in fact more inclined to externalize their prior offenses (Mossière et al., 2020; Taylor et al., 2021). Community participants, in contrast, might rather compare themselves to their non-criminal peers, which makes it more difficult for them to externalize their misconduct and results in a self-image of being at least as antisocial as their peers. This could explain the differential effects in Study 1 and Study 3a (i.e., online vs. mixed sample).

Although psychopathy is associated with a higher self-esteem (Hare, 2003), however, it is still unclear whether highly psychopathic individuals indeed have a more positively valenced self-concept. Whereas some studies suggest that they rate themselves more favorably regarding certain personality traits (e.g., Taylor et al., 2021), other studies suggest that positive (i.e., prosocial) traits and behaviors are rather less central to their self-concept (e.g., van de Groep et al., 2023). As one reviewer pointed out, in addition, highly psychopathic individuals might differ from others with respect to the means they use to protect their positive self-image. Whereas highly psychopathic individuals might rely on antisocial behaviors such as aggression, others might also use more adaptive strategies. Given the unclear empirical evidence as well as the rather weak effect sizes and methodological limitations of our study, successful replications would be needed to draw such strong inferences.

Another possible explanation is that response inhibition deficits hindered highly psychopathic participants to act against the response learned in the compatible block when completing the incompatible block of the aSC-IAT in Study 3a. As no such effects were observed in any of the other studies, however, this interpretation seems rather improbable. Moreover, PCL-R scores in Study 3a were unrelated to difference scores calculated on the basis of error rates (see Supplementary material).

We also controlled for the possibility that highly psychopathic individuals might just be relatively unable to quickly recognize and accurately categorize pictures of social or moral transgressions. In Study 2 and Study 3b, highly psychopathic participants did not perform worse on two simple picture sorting tasks than less psychopathic participants, which speaks against possible effects of picture recognition. As the convergent relationships were not replicated in these studies, however, this alternative explanation needs further examination.

### Practical implications

Another aim of this study was to complement existing psychopathy assessments with two indirect measures. Given the heterogeneous nature of any associations observed, the unsatisfactory stability, as well as the comparatively small incremental contributions provided by the indirect measures (if at all), however, any recommendation for their practical use would be premature. In fact, our findings do not support the practical utility of the SC-IAT.

As these findings might have been affected by either a small sample size (Study 3b; *N* = 47) or a relatively low base of psychopathy (Study 1 to Study 3a), we highly recommend a replication of Study 3b in a larger sample that allows for the comparison of offenders and community participants. In such a study, measures of responsibility, guilt, and externalization (for one example see Mossière et al., 2020), as well as empathy should be included. Until relationships with these measures are established, we cannot be certain that the SC-IATs actually measured empathy and responsibility rather than, for instance, Extraversion. Even if future studies should allow identifying more robust links, the technicalities of the assessment still pose risks for the ecological validity when applying such indirect measures for routine assessment (e.g., potential for measurement error, see Schimmack, 2021). At this stage, we therefore do not recommend to use them for psychopathy assessment.

### Limitations

All four studies suffered from a couple of methodological limitations. On the one hand, the SC-IAT has been criticized for being a less complex task than the customary IAT. Schnabel et al. (2008) have argued that participants could enhance their task performance by simply focusing on the key assigned to one category and pressing the other key for all stimuli that belong to the combined categories. Such effects might be further enhanced by the combination of words representing one and pictures representing the other categories (see Karpinski and Steinman, 2006). In our studies, error-rates below 10% were quite common (more than 87% of the cases). True variance in SC-IAT scores might thus have been overlaid by task switching abilities or faking, resulting in weaker correlations with other measures. Therefore, future studies should increase task difficulty by implementing a response time-out and focusing on word stimuli only.

Second, the choice of target categories needs further consideration. Nosek (2005) showed that implicit-explicit correlations increase to the extent that the target categories represent two extremes of one dimension. The terms *compassion* and *neutral,* however, do not represent antonyms since the absence of compassion is not the exact opposite of compassion. Relationships between the eSC-IAT and the direct measures might thus be more pronounced if more appropriate categories are used (e.g., *compassionate* vs. *callous*/*indifferent*). For replications, the target categories should thus be reconsidered.

Third, the samples drawn from the community (Studies 1 to 3a) and the correctional facilities (Study 3a and Study 3b) varied considerably regarding biological sex and level of education (see Supplementary material). Given the known sex effects in psychopathy (Hollerbach et al., 2020), we cannot rule out the possibility that the differences between Study 1 and Study 3a primarily resulted from subsample differences in these variables. Future studies should therefore more thoroughly match the subsamples regarding sex and education.

Fourth, the internal consistency of the SRP 4 was unsatisfactory in the community samples (e.g., Cronbach’s *α* = 0.56 for the Antisociality subscale in Study 1). As outlined above, this might indicate an overrepresentation of relatively low psychopathy scores in Studies 1 and 2. Given its strong reliance on criminal behavior, on the other hand, the SPR 4 might not be the most adequate measure of the antisociality self-concept in community samples. This seems plausible in the light of item difficulties: Few individuals described themselves as moderately or even highly antisocial, given the overtly criminal content of some of the items in question.

Highly psychopathic individuals in community samples might ascribe other types of antisocial proclivity to themselves, such as cheating, betrayal, or other forms of reckless conduct. Such a different perspective on antisociality in correctional and community samples may also have accounted for the differential results in the aSC-IAT, in which antisociality was inferred from a self-concept as being criminal (i.e., combination of me and legal). Thus, both interpretations may explain the observed differences between community and correctional samples. Therefore, the SRP 4 should be supplemented with other self-report measures and implemented in more heterogeneous samples in potential replication studies.

Only small to moderate correlations were observed between the SC-IATs and the other measures. This is plausible, as we had expected the different types of measures to be differentially affected by spontaneous and deliberate mental processes and by self-presentational attempts. For the SC-IATs, however, these *a priori* assumptions have to be tested empirically by implementing measures of socially desirable responding and automatic information-processing. It was beyond the scope of this research, but potential replications of Study 3a and 3b should also collect information on the type and severity of index offenses and the duration of imprisonment as these variables might influence the implicit self-concept as well as self-control and executive functioning.

## Conclusion

Highly psychopathic individuals are often described as emotionally indifferent and antisocial. These labels, however, primarily reflect how others perceive them. The results of our studies suggest that at the implicit level, the notion of lower emotional involvement/higher antisocial propensity applies to individuals sampled from the community only. Highly psychopathic offenders, in contrast, might have perceived themselves as less antisocial compared to other offenders. This dissociation was not consistently observed across all three studies. Moreover, test–retest correlations of the measurement outcomes were insufficient. The potential of the (SC)-IAT for clinical purposes is not supported by the extant results.

## Data availability statement

The datasets presented in this study can be found in online repositories and retrieved via the following doi: https://doi.org/10.17605/OSF.IO/EMD8Y.

## Ethics statement

All four study protocols were approved by either the ethics committee of the German Psychological Association (DGPs), the local ethics representative/ethics committee of the University of Hagen, or both. The studies were conducted in accordance with the local legislation and institutional requirements. The participants provided their written informed consent to participate in this study.

## Author contributions

JK: Conceptualization, Formal analysis, Investigation, Methodology, Project administration, Visualization, Writing – original draft, Writing – review & editing. DY: Conceptualization, Investigation, Methodology, Validation, Writing – review & editing. KZ: Investigation, Methodology, Writing – review & editing. FB: Investigation, Resources, Writing – review & editing. AM: Conceptualization, Resources, Supervision, Writing – review & editing.

## Footnotes

^1^Part of the data reported in this research (e.g., some PCL-R and SRP 4 scores) have already been reported in other studies (e.g., Brunner et al., 2019; Hollerbach et al., 2020; Hauser et al., 2021). Still, this is the only instance in which the SC-IAT data were analyzed. Furthermore, results regarding some of the preregistered hypotheses will not be described in this but in other publications.

^2^ https://doi.org/10.17605/OSF.IO/EMD8Y

^3^Note that hypothesis 1 and the respective sub-hypotheses were originally preregistered as undirected hypotheses. As negative relationships between the SC-IATs and the SRP 4 were more plausible for theoretical and empirical reasons, the final hypotheses tested in Study 1 were directional.

^4^Note that the antisociality-SC-IAT was erroneously labeled as sociality-SC-IAT in the preregistration forms for Study 2 and Study 3b.

^5^Owing to technical problems, data on age was only available for 153 (total sample) and 66 (offender sample) participants, respectively.

^6^Note that in Germany forensic-psychiatric hospitals not only cater for patients deemed not guilty due to insanity pleas but to patients diagnosed with severe personality disorder as well.

^7^For the PCL-R, ICC was estimated for 36 cases that were scored by two raters based on either the same interview or separate interviews performed by each rater. For the PCL:SV, ICC was estimated based on 13 cases that were scored by two raters based on the same interview.
