# Supplementary material for: In the I of the beholder: an attempt to capture the implicit self-concept regarding psychopathy
Source: Front Psychol. 2024 Jun 17;15:1346029. doi: 10.3389/fpsyg.2024.1346029 (PMC11216285; doi:10.3389/fpsyg.2024.1346029)
Supplement: Supplementary file 1 [file Data_Sheet_1.docx]

**Online Supplementary material**

This document provides a more detailed description of the methodology, data preparation as well as preliminary and additional analyses. This information might be helpful if the readers want to gain a deeper understanding of sample characteristics, procedure, data preparation and exclusions, as well as the robustness of the results. In addition, the supplementary tables and figures visualize the results and provide additional information.

**Table S1**

*Descriptive statistics, reliabilities and intercorrelations for dependent variables in Study 2 (t_1_).*

|  | *M* | *SD* | *Rel* | 2 | 3 | 4 | 5 | 6 | 7 | 8 | 9 | | 10 | | 11 | | 12 | | 13 |
| --- | --- | --- | --- | --- | --- | --- | --- | --- | --- | --- | --- | --- | --- | --- | --- | --- | --- | --- | --- |
| 1. aSC-IAT | -0.12 | 0.32 | .78 ^d^ | .07^a^ | -.02 ^b^ | .06 ^b^ | .09 ^b^ | .07 ^b^ | .09 ^b^ | .04 ^b^ | -.05 ^b^ | -.06 ^b^ | | -.02 ^b^ | | .03 ^b^ | | -.11 ^bc^ | |
| 2. eSC-IAT | 0.11 | 0.35 | .80 ^d^ |  | -.04 ^a^ | .01 ^a^ | .90 ^a†^ | .01 ^a^ | .06 ^b^ | -.01 ^b^ | .02 ^b^ | -.10 ^b^ | | .06 ^b^ | | -.03 ^b^ | | .01 ^bc^ | |
| 3. SRP 4 total | 127.30 | 22.83 | .87 ^e^ |  |  | -.53 ^b^** | -.35 ^b^** | .07 ^b^ | -.41 ^b^** | -.14 ^b^* | .38 ^b^** | .23 ^b^** | | .10 ^b^ | | .48 ^b^** | | .45 ^bc^** | |
| 4. HEX HH | 36.79 | 7.09 | .75 ^e^ |  |  |  | .05 ^b^ | .00 ^b^ | .21 ^b^** | .12 ^b†^ | -.21 ^b^** | -.70 ^b^ | | -.04 ^b^ | | -.28 ^b^** | | -.35 ^bc^** | |
| 5. HEX EM | 31.48 | 6.18 | .71 ^e^ |  |  |  |  | -.21 ^b^** | -.11 ^b^ | -.06 ^b^ | .25 ^b^** | -.03 ^b^ | | .16 ^b^* | | -.39 ^b^** | | -.06 ^bc^ | |
| 6. HEX EX | 33.54 | 6.56 | .74 ^e^ |  |  |  |  |  | .12 ^b†^ | -.05 ^b^ | -.12 ^b†^ | .21 ^b^** | | -.17 ^b^** | | .34 ^b^** | | -.07 ^bc^ | |
| 7. HEX A | 31.63 | 6.64 | .78 ^e^ |  |  |  |  |  |  | .05 ^b^ | -.43 ^b^** | -.23 ^b^** | | -.10 ^b†^ | | -.06 ^b^ | | -.34 ^bc^** | |
| 8. HEX C | 37.32 | 5.83 | .74 ^e^ |  |  |  |  |  |  |  | -.39 ^b^** | -.56 ^b^** | | -.68 ^b^** | | -.06 ^b^ | | -.14 ^bc^* | |
| 9. UPPS U | 30.42 | 8.28 | .88 ^e^ |  |  |  |  |  |  |  |  | .45 ^b^** | | .50 ^b^** | | .12 ^b†^ | | .42 ^bc^** | |
| 10. UPPS PRE | 24.82 | 5.66 | .82 ^e^ |  |  |  |  |  |  |  |  |  | | .44 ^b^** | | .28 ^b^** | | .17 ^bc^** | |
| 11. UPPS PER | 21.42 | 5.21 | .81 ^e^ |  |  |  |  |  |  |  |  |  | |  | | -.06 ^b^ | | .21 ^bc^** | |
| 12. UPPS SES | 35.99 | 8.89 | .85 ^e^ |  |  |  |  |  |  |  |  |  | |  | |  | | .14 ^bc^* | |
| 13. ANTIQUE | 17.84 | 3.89 | .70 ^e^ |  |  |  |  |  |  |  |  |  | |  | |  | |  | |

*Notes*. *N* =219. Rel = reliability. aSC-IAT = Antisociality Single Category Implicit Association Test. eSC-IAT = Empathy Single Category Implicit Association Test. SRP 4 total = Self-Report Psychopathy Scale total score. HEX HH = HEXACO-60 Honesty-Humility Scale. HEX EM = HEXACO-60 Emotionality Scale. HEX EX = HEXACO-60 Extraversion Scale. HEX A = HEXACO-60 Agreeableness Scale. HEX C = HEXACO-60 Conscientiousness Scale. UPPS U = UPPS Urgency Scale. UPPS PRE = UPPS Premeditation Scale. UPPS PER = UPPS Persistence Scale. UPPS SES = UPPS Sensation Seeking Scale. ANTIQUE = Antisocial Behavior Questionnaire.

^a^ one-sided significance testing. ^b^ two-sided significance testing. ^c^ Spearman’s rho. ^d^ *r*_tt_. ^e^ α.

** *p* <.01. * *p* <.05. † *p* < .10.

**Table S2**

*Reliabilities and Intercorrelations for all dimensional measures completed in Study 2 (t_1_).*

|  | *Rel* | 2 | 3 | 4 | 5 | 6 | 7 | 8 | 9 | 10 | 11 | 12 | 13 | 14 | 15 | 16 | 17 |
| --- | --- | --- | --- | --- | --- | --- | --- | --- | --- | --- | --- | --- | --- | --- | --- | --- | --- |
| 1. aSC-IAT | .78 ^d^ | .07^a^ | -.02 ^b^ | .06 ^b^ | .09 ^b^ | .07 ^b^ | .09 ^b^ | .04 ^b^ | -.05 ^b^ | -.06 ^b^ | -.02 ^b^ | .03 ^b^ | -.11 ^bc^ | -.03 ^b^ | .03 ^b^ | -.03 ^b^ | -.06 ^b^ |
| 2. eSC-IAT | .80 ^d^ |  | -.04 ^a^ | .01 ^a^ | .90 ^a†^ | .01 ^a^ | .06 ^b^ | -.01 ^b^ | .02 ^b^ | -.10 ^b^ | .06 ^b^ | -.03 ^b^ | .01 ^bc^ | .14 ^b^* | -.06 ^b^ | -.16 ^b^* | -.11 ^b^ |
| 3. SRP 4 total | .87 ^e^ |  |  | -.53 ^b^** | -.35 ^b^** | .07 ^b^ | -.41 ^b^** | -.14 ^b^* | .38 ^b^** | .23 ^b^** | .10 ^b^ | .48 ^b^** | .45 ^bc^** | -.12 ^b†^ | -.05 ^b^ | -.20 ^b^** | -.13 ^b†^ |
| 4. HEX HH | .75 ^e^ |  |  |  | .05 ^b^ | .00 ^b^ | .21 ^b^** | .12 ^b†^ | -.21 ^b^** | -.70 ^b^ | -.04 ^b^ | -.28 ^b^** | -.35 ^bc^** | .08 ^b^ | -.01 ^b^ | .27 ^b^** | .10 ^b^ |
| 5. HEX EM | .71 ^e^ |  |  |  |  | -.21 ^b^** | -.11 ^b^ | -.06 ^b^ | .25 ^b^** | -.03 ^b^ | .16 ^b^* | -.39 ^b^** | -.06 ^bc^ | .08 ^b^ | .09 ^b^ | -.10 ^b^ | -.01 ^b^ |
| 6. HEX EX | .74 ^e^ |  |  |  |  |  | .12 ^b†^ | -.05 ^b^ | -.12 ^b†^ | .21 ^b^** | -.17 ^b^** | .34 ^b^** | -.07 ^bc^ | .04 ^b^ | .04 ^b^ | -.01 ^b^ | -.02 ^b^ |
| 7. HEX A | .78 ^e^ |  |  |  |  |  |  | .05 ^b^ | -.43 ^b^** | -.23 ^b^** | -.10 ^b†^ | -.06 ^b^ | -.34 ^bc^** | -.03 ^b^ | .07 ^b^ | .10 ^b^ | .02 ^b^ |
| 8. HEX C | .74 ^e^ |  |  |  |  |  |  |  | -.39 ^b^** | -.56 ^b^** | -.68 ^b^** | -.06 ^b^ | -.14 ^bc^* | .04 ^b^ | .17 ^b^* | -.06 ^b^ | .12 ^b†^ |
| 9. UPPS U | .88 ^e^ |  |  |  |  |  |  |  |  | .45 ^b^** | .50 ^b^** | .12 ^b†^ | .42 ^bc^** | -.08 ^b^ | -.10 ^b^ | .12 ^b†^ | -.09 ^b^ |
| 10. UPPS PRE | .82 ^e^ |  |  |  |  |  |  |  |  |  | .44 ^b^** | .28 ^b^** | .17 ^bc^** | -.06 ^b^ | -.16 ^b^* | .03 ^b^ | -.04 ^b^ |
| 11. UPPS PER | .81 ^e^ |  |  |  |  |  |  |  |  |  |  | -.06 ^b^ | .21 ^bc^** | -.05 ^b^ | -.11 ^b^ | -.05 ^b^ | -.11 ^b^ |
| 12. UPPS SES | .85 ^e^ |  |  |  |  |  |  |  |  |  |  |  | .14 ^bc^* | -.01 ^b^ | -.14 ^b^* | -.25 ^b^** | -.10 ^b^ |
| 13. ANTIQUE | .70 ^e^ |  |  |  |  |  |  |  |  |  |  |  |  | -.05 ^bc^ | .03 ^bc^ | -.18 ^bc^** | -.10 ^bc^ |
| 14. eCT | .74 ^d^ |  |  |  |  |  |  |  |  |  |  |  |  |  | .04 ^b^ | -.06 ^b^ | -.03 ^b^ |
| 15. aCT | .66 ^d^ |  |  |  |  |  |  |  |  |  |  |  |  |  |  | -.11 ^b^ | .02 ^b^ |
| 16. age | - |  |  |  |  |  |  |  |  |  |  |  |  |  |  |  | .29 ^b^** |
| 17. education | - |  |  |  |  |  |  |  |  |  |  |  |  |  |  |  |  |

**Table S2 (continued)**

*Notes*. *N* = 219. Rel = reliability. aSC-IAT = Antisociality Single Category Implicit Association Test. eSC-IAT = Empathy Single Category Implicit Association Test. SRP 4 total = Self-Report Psychopathy Scale total score. HEX HH = HEXACO-60 Honesty-Humility Scale. HEX EM = HEXACO-60 Emotionality Scale. HEX EX = HEXACO-60 Extraversion Scale. HEX A = HEXACO-60 Agreeableness Scale. HEX C = HEXACO-60 Conscientiousness Scale. UPPS U = UPPS Urgency Scale. UPPS PRE = UPPS Premeditation Scale. UPPS PER = UPPS Persistence Scale. UPPS SES = UPPS Sensation Seeking Scale. ANTIQUE = Antisocial Behavior Questionnaire. aCT = Antisociality Categorization Task. eCT = Empathy Categorization Task. education = level of education.

^a^ one-sided significance testing. ^b^ two-sided significance testing. ^c^ Spearman’s rho. ^d^ *r*_tt_. ^e^ α

** *p* <.01. * *p* <.05. † *p* < .10

**Figure S1**

*Test-retest differences in SC-IAT D-Scores as a function of mean D-Scores over both measurement occasions in Study 2 for each participant.*

*Note.* *N* = 110. Black lines represent the average test-retest difference (bias), grey lines represent the upper and lower limits of 95% reference intervals around the bias. Upper plot: antisociality SC-IAT (aSC-IAT). Lower plot: empathy SC-IAT (eSC-IAT).

**Table S3**

*Estimates of absolute and relative stability of the SC-IATs in Study 2.*

|  | t_1_ | | |  | t_2_ | | | |  | |  | |  | | Bland-Altman method | | | | | | | | | | |  |
| --- | --- | --- | --- | --- | --- | --- | --- | --- | --- | --- | --- | --- | --- | --- | --- | --- | --- | --- | --- | --- | --- | --- | --- | --- | --- | --- |
|  | *M* | *SD* | *Rel* |  | *M* | *SD* | *Rel* | |  | | *r_tt_*  [95% CI] | |  | | bias  [95% CI] | | *SD*_bias_ | | *t* | | *p* | | 95% LOA LL  [95% CI] | | 95% LOA UL  [95% CI] |  |
| eSC-IAT | 0.14 | 0.35 | .82 |  | 0.09 | 0.39 | .84 |  | | .40 [.26, 1.0] | |  | | 0.04 [-0.04, 0.12] | | 0.40 | | 1.06 | | .291 | | -0.75 [-0.88, -0.62] | | 0.83 [0.70, 0.96] | | |
| aSC-IAT | -0.09 | 0.33 | .78 |  | -0.03 | 0.33 | .77 |  | | .31 [.16, 1.0] | |  | | -0.07 [-0.14, 0.00] | | 0.39 | | -1.86 | | .065 | | -0.83 [-0.96, -0.71] | | 0.69 [0.57, 0.82] | | |

*Notes.* Structure adapted from Welsch et al. (2021)*. N* = 110 (eSC-IAT). Means, Standard deviations and Reliabilities were calculated for the test-retest samples only. eSC-IAT = Empathy Single Category Implicit Association Test. aSC-IAT = Antisociality Single Category Implicit Association Test.

**Table S4**

*Regression results for the prediction of SRP 4 total scores by aCT and aSC-IAT (upper part) and eCT and eSC-IAT (lower part) in Study 2.*

|  |  |  |  | *Bootstrap* | | | | |  | |  |
| --- | --- | --- | --- | --- | --- | --- | --- | --- | --- | --- | --- |
| step | predictor | *B* | *SE B* | *B* | *SE B* | | *95% CI LL* | *95% CI UL* | *∆R*^2^ | |  |
| 1 | Constant | 127.95** | 1.72 |  |  | |  |  |  | |  |
|  | aCT | -3.45 | 4.63 |  |  | |  |  | -.00 | |  |
| 2 | Constant | 127.79** | 1.81 |  |  | |  |  |  | |  |
|  | aCT | -3.41 | 4.64 |  |  | |  |  |  | |  |
|  | aSC-IAT | -1.28 | 4.64 |  |  | |  |  | .00 | |  |
| 1 | Constant | 125.26** | 1.89 | 125.26** | | 1.80 | 121.77 | 128.83 | |  | |
|  | eCT | -7.44^†^ | 4.35 | -7.44^†^ | | 4.11 | -15.64 | 0.56 | | .01 | |
| 2 | Constant | 125.49** | 2.00 | 125.49** | | 1.97 | 121.67 | 129.35 | |  | |
|  | eCT | -7.21 | 4.40 | -7.21^†^ | | 4.19 | -15.34 | 1.42 | |  | |
|  | eSC-IAT | -1.56 | 4.28 | -1.56 | | 4.44 | -9.81 | 7.60 | | .00 | |

*Notes*. *n* = 219. eSC-IAT = Empathy Single-Category Implicit Association Test. aSC-IAT = Antisociality Single-Category Implicit Association Test. aCT = Antisociality Categorization Task. eCT = Empathy Categorization Task. As eCT Scores were non-normally distributed, bootstrapping was applied to the second regression analysis (1,000 draws).

** *p* <.01. * *p* <.05. † *p* < .10.

**Table S5**

*Structure of the double-categorization critical blocks of both SC-IATs.*

|  | block | number of trials | left key | right key |
| --- | --- | --- | --- | --- |
| aSC-IAT | compatible | 72 | self + legal | illegal |
|  | incompatible | 72 | legal | self + illegal |
| eSC-IAT | compatible | 72 | self + compassion | neutral |
|  | incompatible | 72 | compassion | self + neutral |

*Notes.* aSC-IAT = Antisociality Single Category Implicit Association Test. eSC-IAT = Empathy Single Category Implicit Association Test. SC-IAT D-Scores were calculated in terms of differences in mean latencies between the incompatible and the compatible block divided by the pooled standard deviation across both blocks.

**Table S6**

*Stimuli used in the Single Category Implicit Association Tests.*

|  | category | words | pictures |
| --- | --- | --- | --- |
|  | me  [Ich] | my [mein]  I [ich]  me [mich]  self [selbst]  myself [mir] |  |
| eSC-IAT | compassion  [Mitgefühl] |  | 2457  2900.1  3180  3300  9561 |
|  | neutral  [Neutral] |  | 7009  7052  7026  7175  7705 |
| aSC-IAT | legal  [legal] |  | 2550  2655.2  4617  8461  2347 |
|  | illegal  [illegal] |  | 2745.2  2751  6315  6830  9101 |

*Notes*. German expressions in brackets. Numbers represent IAPS catalog numbers. eSC-IAT = Empathy Single Category Implicit Association Test. aSC-IAT = Antisociality Single Category Implicit Association Test.

**Table S7**

*Exemplars of the Stimuli used in the Categorization Tasks.*

| eCT | | |  | aCT | | |
| --- | --- | --- | --- | --- | --- | --- |
| compassion [Mitgefühl] |  | neutral [neutral] |  | legal [legal] |  | illegal [illegal] |
| 2053, 2095, 2141, 2205, 2276, 2278, 2301, 2312, 2455, 2457, 2458, 2661, 2700, 2703, 2799, 2800, 2900.1, 3180, 3181, 3215 |  | 7001, 7002, 7003, 7004, 7006, 7009, 7010, 7012, 7017, 7021, 7025, 7026, 7035, 7040, 7041, 7045, 7052, 7053, 7055, 7059 |  | 2158, 2216, 2299, 2339, 2340, 2345, 2347, 2389, 2391, 2389, 2515, 2550, 2560, 2593, 2595, 2655.2, 4100, 4599, 4600 |  | 2683, 2691, 2710, 2716, 2717, 2745.2, 2751, 3500, 3530, 6211, 6312, 6315, 6350, 6360, 6540, 6550, 6560, 6562, 6563 |

*Notes*. German expressions in brackets. Numbers represent IAPS catalog numbers. eCT = Empathy Categorization Task. aCT = Antisociality Categorization Task. Other pictures were taken from the IAPS and a free picture database. IAPS numbers and links can be shared upon request.

**Table S8**

*Zero-order correlations between all instruments implemented in Study 3a.*

|  | *M* | *SD* | α/*r*_tt_ | 2 | 3 | 4 | 5 | 6 | 7 | 8 | 9 | 10 | 11 | 12 | 13 | 14 |  |
| --- | --- | --- | --- | --- | --- | --- | --- | --- | --- | --- | --- | --- | --- | --- | --- | --- | --- |
| 1. eSC-IAT | 0.05 | 0.39 | .90 | .16 ^b^* | -.09 ^b^ | -.09 ^b^ | -.07 ^b^ | -.12 ^b†^ | -.09 ^b^ | -.02 ^b^ | -.04 ^b^ | -.06 ^b^ | .06 ^b^ | -.03 ^b^ | .01 ^d^ | -.02 ^b^ | |
| 2. aSC-IAT | 0.03 | 0.31 | .74 |  | .10 ^b^ | .15 ^b^* | .09 ^b^ | .15 ^b^* | .06 ^b^ | .00 ^b^ | -.01 ^b^ | .00 ^b^ | .01 ^b^ | .01 ^b^ | -.08 ^d^ | .08 ^b^ | |
| 3. PCL-R total | 12.05 | 11.36 | - |  |  | .77 ^a^** | .91 ^a^** | .83 ^a^** | .82 ^a^** | .72 ^a^** | .36 ^a^** | .54 ^a^** | .51 ^a^** | .81 ^a^** | -.44 ^c^** | -.04 ^b^ | |
| 4. PCL-R INT | 2.45 | 2.38 | - |  |  |  | .69 ^a^** | .54 ^a^** | .48 ^a^** | .50 ^a^** | .39 ^a^** | .42 ^a^** | .35 ^a^** | .45 ^a^** | -.17 ^c†^ | .01 ^b^ | |
| 5. PCL-R AFF | 2.80 | 3.01 | - |  |  |  |  | .71 ^a^** | .70 ^a^** | .57 ^a^** | .25 ^a^** | .44 ^a^** | .35 ^a^** | .71 ^a^** | -.38 ^c^** | -.02 ^b^ | |
| 6. PCL-R LIF | 3.08 | 3.22 | - |  |  |  |  |  | .82 ^a^** | .62 ^a^** | .24 ^a^** | .43 ^a^** | .49 ^a^** | .74 ^a^** | -.37 ^c^** | -.03 ^b^ | |
| 7. PCL-R ANT | 2.65 | 3.36 | - |  |  |  |  |  |  | .70 ^a^** | .29 ^a^** | .47 ^a^** | .53 ^a^** | .83 ^a^** | -.45 ^c^** | -.07 ^b^ | |
| 8. SRP 4 total | 147.74 | 35.49 | .94 |  |  |  |  |  |  |  | .75 ^a^** | .82 ^a^** | .87 ^a^** | .82 ^a^** | -.29 ^c^** | -.12 ^b†^ | |
| 9. SRP 4 INT | 38.12 | 9.29 | .84 |  |  |  |  |  |  |  |  | .60 ^a^** | .65 ^a^** | .35 ^a^** | -.08 ^c^ | -.13 ^b†^ | |
| 10. SRP 4 AFF | 35.77 | 9.02 | .82 |  |  |  |  |  |  |  |  |  | .63 ^a^** | .53 ^a^** | -.12 ^c^ | -.15 ^b^* | |
| 11. SRP 4 LIF | 42.12 | 10.51 | .83 |  |  |  |  |  |  |  |  |  |  | .59 ^a^** | -.15 ^c†^ | -.13 ^b†^ | |
| 12. SRP 4 ANT | 31.73 | 14.69 | .90 |  |  |  |  |  |  |  |  |  |  |  | -.45 ^c^** | -.03 ^b^ | |
| 13. WMT-2 | 10.61 | 4.18 | .83 |  |  |  |  |  |  |  |  |  |  |  |  | -.06 ^c^ | |
| 14. cSC-IAT | 0.15 | 0.30 | .75 |  |  |  |  |  |  |  |  |  |  |  |  |  | |

**Table S8 (continued)**

*Notes*. eSC-IAT = empathy Single-Category Implicit Association Test. aSC-IAT = antisociality Single-Category Implicit Association Test. PCL-R total = Psychopathy Checklist-Revised total score. PCL-R INT = Psychopathy Checklist-Revised Interpersonal facet. PCL-R AFF = Psychopathy Checklist-Revised Affective facet. PCL-R LIF = Psychopathy Checklist-Revised Lifestyle facet. PCL-R ANT = Psychopathy Checklist-Revised Antisocial facet. SRP 4 total = Self-Report Psychopathy Scale total score. SRP 4 INT = Self-Report Psychopathy Scale Interpersonal scale. SRP 4 AFF = Self-Report Psychopathy Scale Affective scale. SRP 4 LIF = Self-Report Psychopathy Scale Lifestyle scale. SRP 4 ANT = Self-Report Psychopathy Scale Antisocial behavior scale. WMT-2 = Wiener Matrizen Test 2. cSC-IAT = control Single Category Implicit Association Test.

^a^ *n* = 166. ^b^ *n* = 162. ^c^ *n* = 127. ^d^ *n* = 123.

** *p* <.01. * *p* <.05. † *p* < .10.

**Table S9**

*SRP 4 total and facet scores and SC-IAT scores regressed on PCL-R total and facet scores in Study 3a.*

|  |  |  | PCL-R total | | |  | PCL-R INT | | |  | PCL-R AFF | | |  | PCL-R LIF | | |  | PCL-R ANT | | |
| --- | --- | --- | --- | --- | --- | --- | --- | --- | --- | --- | --- | --- | --- | --- | --- | --- | --- | --- | --- | --- | --- |
| Step | Predictors |  | *β* | *t* | *ΔR^2^* |  | *β* | *t* | *ΔR^2^* |  | *β* | *t* | *ΔR^2^* |  | *β* | *t* | *ΔR^2^* |  | *β* | *t* | *ΔR^2^* |
| 1 | corresponding SRP 4 score |  | .71 | 12.67** | .50** |  | .37 | 5.00** | .13** |  | .44 | 6.17** | .19** |  | .48 | 6.86** | .23** |  | .83 | 18.55** | .68** |
| 2 | corresponding SRP 4 score |  | .71 | 12.79** |  |  | .37 | 5.02** |  |  | .44 | 6.11** |  |  | .49 | 7.17** |  |  | .82 | 18.66** |  |
|  | aSC-IAT |  | .11 | 2.00* |  |  | .16 | 2.24* |  |  | .10 | 1.33 |  |  | .17 | 2.47* |  |  | .07 | 1.61 |  |
|  | eSC-IAT |  | -.10 | -1.75^†^ | .02^†^ |  | -.10 | -1.33 | .03^†^ |  | -.06 | -0.84 | .01 |  | -.18 | -2.58* | .05** |  | -.08 | -1.80^†^ | .01^†^ |

*Notes*. *n* = 162. PCL-R total = Psychopathy Checklist-Revised total score. PCL-R INT = Psychopathy Checklist-Revised Interpersonal facet. PCL-R AFF = Psychopathy Checklist-Revised Affective facet. PCL-R LIF = Psychopathy Checklist-Revised Lifestyle facet. PCL-R ANT = Psychopathy Checklist-Revised Antisocial facet. Corresponding SRP 4 score = corresponding score on the Self Report Psychopathy Scale 4^th^ ed. eSC-IAT = empathy Single-Category Implicit Association Test. aSC-IAT = antisociality Single-Category Implicit Association Test. Adjusted *R*² reported for step 1.

** *p* < .01. * *p* < .05. ^†^ *p* < .10.

**Table S10**

*Zero-order correlations in the offender sample (below the diagonal) and the community sample (above the diagonal) in Study 3a.*

|  | *M* | *SD* | 1 | 2 | 3 | 4 | | 5 | | 6 | | 7 | | 8 | | 9 | | 10 | | 11 | | 12 | | *M* | | *SD* | |
| --- | --- | --- | --- | --- | --- | --- | --- | --- | --- | --- | --- | --- | --- | --- | --- | --- | --- | --- | --- | --- | --- | --- | --- | --- | --- | --- | --- |
| 1. eSC-IAT | 0.03 | 0.44 |  | .20 ^b†^ | -.01 ^b^ | | -.14 ^b^† | | .13 ^b^ | | .07 ^b^ | | .04 ^b^ | | -.10 ^b^ | | -.12 ^b^ | | -.12 ^b^ | | -.01 ^b^ | | -.06 ^b^ | | 0.07 | | 0.33 |
| 2. aSC-IAT | 0.02 | 0.33 | .14 ^a^ |  | .09 ^b^ | | .07 ^b^ | | .03 ^b^ | | .14 ^b^ | | -.05 ^b^ | | .02 ^b^ | | -.00 ^b^ | | .03 ^b^ | | .06 ^b^ | | -.04 ^b^ | | 0.03 | | 0.29 |
| 3. PCL-R total | 21.26 | 9.31 | -.10 ^a^ | .22 ^a^* |  | | .87 ^d^** | | .69 ^d^** | | .68 ^d^** | | .44 ^d^** | | .40 ^d^** | | .36 ^d^** | | .27 ^d^* | | .38 ^d^** | | .21 ^d^* | | 3.69 | | 4.51 |
| 4. PCL-R INT | 3.56 | 2.35 | -.02 ^a^ | .25 ^a^* | .73 ^c^** | |  | | .52 ^d^** | | .37 ^d^** | | .21 ^d^* | | .37 ^d^** | | .38 ^d^** | | .30 ^d^** | | .29 ^d^** | | .13 ^d^ | | 1.44 | | 1.92 |
| 5. PCL-R AFF | 5.19 | 2.52 | -.11 ^a^ | .22 ^a^* | .81 ^c^** | | .65 ^c^** | |  | | .23 ^d^* | | .04 ^d^ | | .22 ^d^* | | .21 ^d†^ | | .26 ^d^* | | .10 ^d^ | | .11 ^d^ | | 0.63 | | 1.25 |
| 6. PCL-R LIF | 5.57 | 2.77 | -.20 ^a^* | .29 ^a^* | .59 ^c^** | | .34 ^c^** | | .36 ^c^** | |  | | .47 ^d^** | | .24 ^d^* | | .17 ^d^ | | .02 ^d^ | | .38 ^d^** | | .15 ^d^ | | 0.82 | | 1.42 |
| 7. PCL-R ANT | 5.27 | 3.17 | -.10 ^a^ | .17 ^a†^ | .62 ^c^** | | .28 ^c^* | | .36 ^c^** | | .64 ^c^** | |  | | .29 ^d^** | | .13 ^d^ | | .05 ^d^ | | .40 ^d^** | | .38 ^d^** | | 0.27 | | 0.71 |
| 8. SRP 4 total | 169.33 | 34.47 | .09 ^a^ | .01 ^a^ | .57 ^c^** | | .30 ^c^** | | .26 ^c^* | | .39 ^c^** | | .56 ^c^** | |  | | .85 ^d^** | | .77 ^d^** | | .83 ^d^** | | .64 ^d^** | | 128.14 | | 22.84 |
| 9. SRP 4 INT | 39.57 | 9.70 | .03 ^a^ | -.01 ^a^ | .43 ^c^** | | .35 ^c^** | | .23 ^c^* | | .22 ^c†^ | | .36 ^c^** | | .83 ^c^** | |  | | .55 ^d^** | | .60 ^d^** | | .38 ^d^** | | 36.80 | | 8.74 |
| 10. SRP 4 AFF | 39.25 | 9.41 | .02 ^a^ | -.01 ^a^ | .51 ^c^** | | .30 ^c^** | | .28 ^c^* | | .35 ^c^** | | .41 ^c^** | | .81 ^c^** | | .62 ^c^** | |  | | .46 ^d^** | | .35 ^d^** | | 32.61 | | 7.39 |
| 11. SRP 4 LIF | 46.09 | 11.45 | .14 ^a^ | -.02 ^a^ | .40 ^c^** | | .19 ^c^ | | .13 ^c^ | | .34 ^c^** | | .48 ^c^** | | .89 ^c^** | | .69 ^c^** | | .64 ^c^** | |  | | .49 ^d^** | | 38.52 | | 8.09 |
| 12. SRP 4 ANT | 44.42 | 11.00 | .08 ^a^ | .06 ^a^ | .55 ^c^** | | .19 ^c^ | | .25 ^c^* | | .39 ^c^** | | .60 ^c^** | | .78 ^c^** | | .46 ^c^** | | .49 ^c^** | | .60 ^c^** | |  | | 20.21 | | 4.68 |

*Notes*. eSC-IAT = empathy Single-Category Implicit Association Test. aSC-IAT = antisociality Single-Category Implicit Association Test. PCL-R total = Psychopathy Checklist-Revised total score. PCL-R INT = Psychopathy Checklist-Revised Interpersonal facet. PCL-R AFF = Psychopathy Checklist-Revised Affective facet. PCL-R LIF = Psychopathy Checklist-Revised Lifestyle facet. PCL-R ANT = Psychopathy Checklist-Revised Antisocial facet.

**Table S10 (continued)**

SRP 4 total = Self-Report Psychopathy Scale total score. SRP 4 INT = Self-Report Psychopathy Scale Interpersonal scale. SRP 4 AFF = Self-Report Psychopathy Scale Affective scale. SRP 4 LIF = Self-Report Psychopathy Scale Lifestyle scale. SRP 4 ANT = Self-Report Psychopathy Scale Antisocial behavior scale.

^a^ *n* = 76. ^b^ *n* = 86. ^c^ *n* = 79. ^d^ *n* = 87.

** *p* <.01. * *p* <.05. † *p* < .10.

**Table S11**

*Zero-order correlations between the SC-IATs, the WMT-2 and all questionnaires implemented in Study 3b.*

|  | *M* | *SD* | *Rel* | 2 | 3 | 4 | 5 | 6 | 7 | 8 | 9 | 10 | 11 | 12 | | 13 | 14 | 15 |
| --- | --- | --- | --- | --- | --- | --- | --- | --- | --- | --- | --- | --- | --- | --- | --- | --- | --- | --- |
| 1. eSC-IAT | 0.09 | 0.38 | .85 | -.21 ^bf^ | .13 ^be^ | -.01 ^be^ | .20 ^be^ | -.18 ^bf^ | .13 ^bf^ | -.19 ^bf^ | -.01 ^bf^ | .02 ^bf^ | -.23 ^bf^ | | -.36 ^bf^* | .30 ^bf†^ | .21 ^bf^ | -.17 ^df^ |
| 2. aSC-IAT | 0.04 | 0.36 | .83 |  | -.39 ^bf^* | .13 ^bf^ | -.13 ^bf^ | .22 ^bf^ | -.09 ^bf^ | -.18 ^bf^ | -.05 ^bf^ | -.14 ^bf^ | .04 ^bf^ | | .18 ^bf^ | .07 ^bf^ | -.02 ^bf^ | .06 ^df^ |
| 3. HEXACO EM | 31.09 | 6.46 | .81 |  |  | -.21 ^af^ | .20 ^af^ | -.13 ^af^ | .33 ^af^* | .08 ^af^ | -.11 ^af^ | .06 ^af^ | -.36 ^af^* | | -.47 ^af^** | -.21^af^ | -.24 ^af^ | .02 ^cf^ |
| 4. HEXACO EX | 31.47 | 6.40 | .78 |  |  |  | -.20 ^af^ | .32 ^af^* | .22 ^af^ | -.35 ^af^ | -.14 ^af^ | -.30 ^af†^ | .31 ^af^* | | .09 ^af^ | .30 ^af†^ | .11 ^af^ | .06 ^cf^ |
| 5. HEXACO HH | 32.40 | 7.83 | .84 |  |  |  |  | .09 ^af^ | .17 ^af^ | -.29 ^af†^ | -.11 ^af^ | -.30 ^af†^ | -.51 ^af^** | | -.33 ^af^* | .05 ^af^ | .02 ^af^ | .20 ^cf^ |
| 6. HEXACO C | 35.49 | 5.89 | .79 |  |  |  |  |  | .19 ^af^ | -.59 ^af^** | -.62 ^af^** | -.72 ^af^** | -.12 ^af^ | | -.09 ^af^ | -.01 ^af^ | -.16 ^af^ | .11 ^cf^ |
| 7. HEXACO A | 30.00 | 5.15 | .68 |  |  |  |  |  |  | -.44 ^af^** | -.33 ^af^* | -.19 ^af^ | -.04 ^af^ | | -.45 ^af^** | -.20 ^af^ | -.24 ^af^ | .19 ^cf^ |
| 8. UPPS U | 36.23 | 9.17 | .90 |  |  |  |  |  |  |  | .67 ^af^** | .54 ^af^** | .34 ^af^* | | .31 ^af^* | -.08 ^af^ | .19 ^af^ | -.27 ^cf†^ |
| 9. UPPS Pre | 27.30 | 6.75 | .88 |  |  |  |  |  |  |  |  | .59 ^af^** | .39 ^af^** | | .10 ^af^ | .03 ^af^ | .17 ^af^ | -.24 ^cf^ |
| 10. UPPS Per | 22.33 | 6.05 | .88 |  |  |  |  |  |  |  |  |  | .12 ^af^ | | .08 ^af^ | -.17 ^af^ | -.03 ^af^ | -.21 ^cf^ |
| 11. UPPS SES | 36.09 | 9.28 | .86 |  |  |  |  |  |  |  |  |  |  | | .32 ^af^* | .19 ^af^ | .27 ^af†^ | -.27 ^cf†^ |
| 12. ANTIQUE | 22.12 | 7.54 | .80 |  |  |  |  |  |  |  |  |  |  | |  | .39 ^af^* | .41 ^af^** | .04 ^cf^ |
| 13. ASB freq | 3.67 | 4.79 | - |  |  |  |  |  |  |  |  |  |  | |  |  | .77 ^af^** | -.11 ^cf^ |
| 14. ASB vers | 1.42 | 1.16 | - |  |  |  |  |  |  |  |  |  |  | |  |  |  | -.25 ^cf^ |
| 15. WMT-2 | 8.58 | 3.73 | .78 |  |  |  |  |  |  |  |  |  |  | |  |  |  |  |

**Table S11 (continued)**

*Notes*. *Rel* = reliability. eSC-IAT = empathy Single-Category Implicit Association Test. aSC-IAT = antisociality Single-Category Implicit Association Test. HEXACO EM = HEXACO-60 Emotionality Scale. HEX EX = HEXACO-60 Extraversion Scale. HEX HH = HEXACO-60 Honesty-Humility Scale. HEX C = HEXACO-60 Conscientiousness Scale. HEX A = HEXACO-60 Agreeableness Scale. UPPS U = UPPS-P Urgency Scale. UPPS PRE = UPPS-P Premeditation Scale. UPPS PER = UPPS-P Perseverance Scale. UPPS SES = UPPS-P Sensation Seeking Scale. ANTIQUE = Antisocial Behavior Questionnaire. ASB freq = Overall number of infractions in the facility. ASB vers = versatility of infractions in the facility. WMT-2 = Wiener Matrizen Test 2.

^a^ *n* = 43. ^b^ *n* = 39. ^c^ *n* = 40. ^d^ *n* = 36. ^e^ one-sided. ^f^ two-sided^.^

** *p* <.01. * *p* <.05. † *p* < .10.

**Table S12**

*Zero-order correlations between the PCL-R scores at in Study 3a, the SC-IAT, CT, and SRP 4 scores in Study 3b.*

|  | *M* | *SD* | *Rel* | 2 | 3 | 4 | 5 | 6 | 7 | 8 | 9 | 10 | 11 | 12 | 13 | 14 |
| --- | --- | --- | --- | --- | --- | --- | --- | --- | --- | --- | --- | --- | --- | --- | --- | --- |
| 1. eSC-IAT | 0.09 | 0.38 | .85 ^d^ | -.21 ^b^ | .07 ^c^ | .01 ^c^ | -.08 ^b^ | -.02 ^b^ | -.05 ^b^ | .05 ^b^ | -.01 ^b^ | -.14 ^b^ | -.17 ^b^ | -.09 ^b^ | -.21 ^b^ | -.01 ^b^ |
| 2. aSC-IAT | 0.04 | 0.36 | .83 ^d^ |  | .13 ^c^ | -.03 ^c^ | .09 ^b^ | -.00 ^b^ | .04 ^b^ | -.04 ^b^ | .18 ^b^ | .23 ^b^ | .28 ^b†^ | .32 ^b^* | .12 ^b^ | .10 ^b^ |
| 3. eCT | -0.39 | 0.39 | .79 |  |  | -.07 ^c^ | .14 ^c^ | .21 ^c^ | .16 ^c^ | -.02 ^c^ | -.00 ^c^ | .00 ^c^ | .02 ^c^ | -.05 ^c^ | .02 ^c^ | .01 ^c^ |
| 4. aCT | 0.16 | 0.29 | .60 |  |  |  | .20 ^c^ | .10 ^c^ | .23 ^c^ | .07 ^c^ | .15 ^c^ | .07 ^c^ | .04 ^c^ | .04 ^c^ | .00 ^c^ | .14 ^c^ |
| 5. PCL-R total | 21.79 | 9.56 | - |  |  |  |  | .77 ^a^** | .80 ^a^** | .52 ^a^** | .57 ^a^** | .55 ^a^** | .40 ^a^** | .49 ^a^** | .30 ^a^** | .53 ^a^** |
| 6. PCL-R INT | 3.65 | 2.45 | - |  |  |  |  |  | .71 ^a^** | .38 ^a^** | .29 ^a†^ | .25 ^a^ | .31 ^a^* | .28 ^a†^ | .20 ^a^ | .08 ^a^ |
| 7. PCL-R AFF | 5.70 | 2.43 | - |  |  |  |  |  |  | .28 ^a†^ | .27 ^a†^ | .17 ^a^ | .24 ^a^ | .19 ^a^ | .01 ^a^ | .16 ^a^ |
| 8. PCL-R LIF | 5.50 | 2.91 | - |  |  |  |  |  |  |  | .58 ^a^** | .28 ^a†^ | .08 ^a^ | .33 ^a^* | .22 ^a^ | .30 ^a†^ |
| 9. PCL-R ANT | 5.12 | 3.40 | - |  |  |  |  |  |  |  |  | .62 ^a^** | .40 ^a^** | .51 ^a^** | .50 ^a^** | .63 ^a^** |
| 10. SRP 4 total | 173.09 | 32.43 | .93 ^e^ |  |  |  |  |  |  |  |  |  | .82 ^a^** | .78 ^a^** | .89 ^a^** | .85 ^a^** |
| 11. SRP 4 INT | 42.14 | 8.38 | .79 ^e^ |  |  |  |  |  |  |  |  |  |  | .63 ^a^** | .64 ^a^** | .54 ^a^** |
| 12. SRP 4 AFF | 40.70 | 8.57 | .78 ^e^ |  |  |  |  |  |  |  |  |  |  |  | .59 ^a^** | .47 ^a^** |
| 13. SRP 4 LIF | 45.95 | 10.06 | .82 ^e^ |  |  |  |  |  |  |  |  |  |  |  |  | .72 ^a^** |
| 14. SRP 4 ANT | 44.30 | 11.74 | .83 ^e^ |  |  |  |  |  |  |  |  |  |  |  |  |  |

**Table S12 (continued)**

*Notes*. *Rel* = reliability. eSC-IAT = Empathy Single category Implicit Association Test. aSC-IAT = antisociality Single-Category Implicit Association Test. eCT = empathy Categorization Task. aCT = antisociality Categorization Task. PCL-R total = Psychopathy Checklist-Revised total score. PCL-R INT = Psychopathy Checklist-Revised Interpersonal facet. PCL-R AFF = Psychopathy Checklist-Revised Affective facet. PCL-R LIF = Psychopathy Checklist-Revised Lifestyle facet. PCL-R ANT = Psychopathy Checklist-Revised Antisocial facet. SRP 4 total = Self-Report Psychopathy Scale total score. SRP 4 INT = Self-Report Psychopathy Scale Interpersonal scale. SRP 4 AFF = Self-Report Psychopathy Scale Affective scale. SRP 4 LIF = Self-Report Psychopathy Scale Lifestyle scale. SRP 4 ANT = Self-Report Psychopathy Scale Antisocial behavior scale.

^a^ *n* = 43. ^b^ *n* = 39. ^c^ *n* = 38. ^c^ *r*_tt_. ^d^ α.

** *p* <.01 (two-sided). * *p* <.05 (two-sided). † *p* < .10.

**Table S13**

*eCT scores and eSC-IAT scores in Study 3b regressed on PCL-R total scores in Study 3a.*

|  |  |  | | | | Bootstrap for coefficients | | | | | |
| --- | --- | --- | --- | --- | --- | --- | --- | --- | --- | --- | --- |
| step | predictor | *B* | *SE B* | *p* | *B* | | *SE B* | *p* | 90% CI LL | 90% CI UL | *∆R*^2^ |
| 1 | Constant | 22.61 | 2.18 | .000 | 22.61 | | 2.13 | .001 | 18.45 | 26.72 |  |
|  | eCT | 3.43 | 4.00 | .397 | 3.43 | | 3.90 | .366 | -4.16 | 11.06 | -.01 |
| 2 | Constant | 22.88 | 2.26 | .000 | 22.88 | | 2.26 | .001 | 18.51 | 27.32 |  |
|  | eCT | 3.58 | 4.05 | .383 | 3.58 | | 3.93 | .359 | -3.86 | 11.24 |  |
|  | eSC-IAT | -2.23 | 4.08 | .588 | -2.23 | | 4.76 | .655 | -12.22 | 6.47 | .01 |

*Notes.* *n* = 38. eSC-IAT = empathy Single-Category Implicit Association Test. eCT = empathy Categorization Task. Adjusted *R*² reported for step 1.

**Table S14**

*aCT scores and aSC-IAT scores in Study 3b regressed on PCL-R total scores in Study 3a.*

|  |  |  | | | | Bootstrap for coefficients | | | | | |
| --- | --- | --- | --- | --- | --- | --- | --- | --- | --- | --- | --- |
| step | predictor | *B* | *SE B* | *p* | *B* | | *SE B* | *p* | 90% CI LL | 90% CI UL | *∆R*^2^ |
| 1 | Constant | 20.24 | 1.73 | .000 | 20.24 | | 1.60 | .001 | 17.03 | 23.42 |  |
|  | aCT | 6.43 | 5.24 | .228 | 6.43 | | 4.92 | .180 | -2.88 | 16.68 | .01 |
| 2 | Constant | 20.24 | 1.75 | .000 | 20.24 | | 1.62 | .001 | 17.06 | 23.51 |  |
|  | aCT | 6.13 | 5.35 | .259 | 6.13 | | 4.94 | .213 | -3.18 | 16.91 |  |
|  | aSC-IAT | 1.83 | 4.28 | .672 | 1.83 | | 4.55 | .680 | -7.06 | 11.28 | .01 |

*Notes.* *n* = 38. aSC-IAT = antisociality Single-Category Implicit Association Test. aCT = antisociality Categorization Task. Adjusted *R*² reported for step 1.

**Additional information on methodology and results**

**Study 1**

**Participants.** 92% of the participants reported to have taken at least the German high school diploma (“Abitur”) in both samples.

**Statistical analyses.** The original data set consisted of 248 participants. 5 participants were excluded owing to missing data regarding age as we could not ensure that they met the inclusion criteria. Another three data sets were removed because of zero intraindividual variance (i.e., *SD* = 0) in the SRP 4. No outliers were detected graphically (i.e., through boxplots). Thirteen participants were excluded due to error-rates above 20% in the eSC-IAT or the aSC-IAT (*n* = 12), or because of a high number of nonresponses in the aSC-IAT (i.e., 49% of the trials; *n* = 1). The final sample thus consisted of 227 participants. A resampling procedure was applied to all non-normally distributed variables in all studies (i.e., Bootstrapping with 1000 draws). For reasons of parsimony, only the results of the parametrical analyses are reported.

**Preliminary analyses.** Prior to the testing of the hypotheses, potential effects of age, sex, education and the experimental order (aSC-IAT first vs. eSC-IAT first) were tested. Significant effects of the order of presentation were neither found for the eSC-IAT (*t*[214.69] = -0.96; *p* = .338) nor for the aSC-IAT (*t*[218.35] = -0.15; *p* = .883). No effects were observed for sex differences in the eSC-IAT (*t*[225] = 1.29, *p* = .199) and the aSC-IAT (*t*[58.08] = -0.45, *p* = .653); for age covariation in the eSC-IAT (*r* = .03, *p* = .606) and the aSC-IAT (*r* = .03, *p* = .652), or for level of education in the eSC-IAT (*rho* = -.02, *p* = .730) and the aSC-IAT (*rho* = .01, *p* = .891). However, men showed significantly higher total SRP 4 scores (*M* = 137.76, *SD* = 24.44) compared to women (*M* = 122.64, *SD* = 23.50) in the present sample (*t*[225] = -3.83, *p* < .001, *d* = 0.64).

**Additional analyses.** When simultaneously entered in a regression analysis, a combination of the eSC-IAT and the aSC-IAT significantly predicted the SRP 4 total score (*Adj. R²* = .03, *p* = .012) as well as the score on the Affective facet (*Adj. R*² = .02, *p* = .023). The other SRP 4 subscale scores were not significantly predicted by a combination of both SC-IATs (*ps* > .05).

**Study 2**

**Participants.** To obtain an estimate of test-retest reliability, corresponding correlations of SC-IAT measures from previous studies (Chevance et al., 2017; Hyde et al., 2017; Nentjes et al., 2017; Stieger, et al., 2010) were subjected to Fisher’s *Z* transformation and aggregated to a mean test-retest-correlation of *r_tt_* = .26 (*k* = 5), an effect size similar to the average IAT-explicit correlation (*r* = .21, *k* = 155) reported by Greenwald et al. (2009). Hence, a small effect size (*r* = .21) was expected prior to the experiment. A minimum sample size of 139 individuals for measurement time point 2 (t_2_) was calculated using G*Power 3.1.9.2 in order to obtain the above-mentioned effect with an actual power of 80% and type I error probability of 5%. The attrition-adjusted targeted sample size was 360 to 420 participants for measurement time point 1 (t_1_), which was larger than the required sample size to test the two-tailed correlations between the SC-IATs and the external criteria (*N* = 175). The final sample at t_1_ consisted of 219 participants at t_1_ (60% female, *M*_age_ = 40.36, *SD*_age_ = 12.74), of whom 15% of the participants reported prior experience with the (SC-)IAT, 90% were right-handed, and 91% had at least the German high-school diploma. At t_2_, the final sample consisted of 110 participants (66% female, *M*_age_ = 41.37, *SD*_age_ = 12.62). 16% of the participants in both t_2_ samples were familiar with the (SC-)IAT, 87% were right-handed, and 90% had at least the German high-school diploma.

**Statistical analyses.** 230 data sets were gathered at t_1_. Owing to technical errors, missing data (response was set to 0) in the SRP 4 was observed for 26 participants at t_1_. Missing values were replaced by the individual mean on the respective subscale (i.e., mean imputation) and data sets with missing values in more than 5% of the items were excluded (*n* = 2 at t_1_, *n* = 0 at t_2_). In sum, six data sets were excluded at t_1_ because of missing data in more than 5% of the items in the SRP 4, because of non-serious participation or zero intraindividual variance in either the SRP 4, the HEXACO-60 or the UPPS. As low base rates were expected regarding antisocial behavior, no plausibility checks were performed on the ANTIQUE. In addition, five data set were removed because of an error-rate above 20% in either the SC-IATs or the CTs.

The original t_2_ sample consisted of 139 participants of whom 27 were excluded as their code for the data match had either not been used at t_1_ (*n* = 8) or twice at t_2_ (*n* = 16), or no match was found at t_1_ (*n* = 3). 112 complete data sets thus remained for the analyses. Of these, 13 participants had missing values in the SRP 4 that were replaced by mean imputation. As prior studies have shown that seriousness checks can improve the validity of study outcomes above and beyond other techniques, participants were asked whether they had participated seriously at the end of both measurement time points (see Aust et al., 2013). Two data sets were removed because of missing data in more than 5% of the items in the SRP 4, non-serious participation, zero intraindividual variance on the questionnaires or error-rates above 20% at either t_1_ or t_2_. The final samples therefore consisted of 219 participants at t_1_ and 110 participants at t_2_. No outliers were observed except for extremely elevated scores on the ANTIQUE. These data sets were not removed with respect to the low base rate of antisociality in community samples. One-tailed testing was used for H1.1. and H1.2, H3.1, H3.2, and H3.3, whereas two-tailed testing was conducted for H2, H4, H5, and the respective sub-hypotheses. Non-parametrical correlations were calculated for the all analyses including the ANTIQUE.

**Preliminary analyses.** Preliminary analyses revealed no significant effects for sex-differences in the eSC-IAT (*t*[222] = -0.32, *p* = .753) and the aSC-IAT (*t*[222] = -1.35, *p* = .178) or for prior experience with the (SC-)IAT (*t*[222] = 0.92, *p* = .359 and *t*[222] = 0.47, *p* = .642, respectively). As previous studies had found worse performance of highly-psychopathic individuals in tasks that require left-hemisphere activation (Glenn & Raine, 2014), handedness was recorded as a potential covariate. Neither the eSC-IAT score (*F*[2, 221] = 0.31, *p* = .733) nor the aSC-IAT score (*F*[2, 221] = 0.84, *p* = .434) were affected by handedness. Significant correlations were observed between participants age and the eSC-IAT score (*r* = -.17, *p* = .012), the SRP 4 total score (*r* = -.22, *p* = .001), HEXACO Honesty-Humility (*r* = .28, *p* < .001), and UPPS Sensation Seeking (*r* = -.27, *p* < .001; see Table S1).

**Additional analyses.** In keeping with Study 1, we tested whether a combination of both SC-IAT scores explained a meaningful amount of variance in the SRP 4 scores at t_1_. This was not the case (all *p*s > .05). Moreover, partial correlations between the eSC-IAT score and the SRP 4 total score as well as HEXACO Honesty-Humility and between the aSC-IAT and the UPPS Sensation Seeking scale were calculated to control for age effects. The results were not significantly affected by participant’s age.

In order to provide more appropriate estimates of absolute stability, the Bland-Altman method (Bland & Altman, 1986, 1999) was applied by plotting test-retest differences in SC-IAT scores against mean SC-IAT scores over both measurements for each participant. Assuming that test-retest differences are normally distributed, the resulting Bland-Altman Plots visualize both average and individual test-retest variability. On the one hand, the average difference in test-retest scores (*bias*) is visualized by a vertical line. For a measure to be stable, this line should intersect the y-axis near zero and thus, the coefficient should not deviate from zero significantly.

Furthermore, Bland-Altman Plots provide an estimate of variability of individual test-retest differences in terms of upper and lower limits of 95% reference intervals around the bias (Limits of Agreement, LOA). Although the terms are not mathematically identical, LOAs can be roughly interpreted in terms of confidence intervals including 95% of the individual test-retest differences observed in the present sample. Hence, larger LOAs imply higher test-retest variability and thus lower temporal stability (see Bland & Altman, 1999). Moreover, individual scores above the upper and below the lower level should be scarce.

Graphic inspection (i.e., QQ-plots and histograms) and Shapiro-Wilk tests indicated that difference scores between t_1_ and t_2_ were normally distributed and not skewed for both SC-IATs. Test-retest differences in the SRP 4 total scores, however, were non-normally distributed (W = 0.97, *p* = .013). Therefore, the Bland-Altman method was applied only to the SC-IAT scores. Following Welsch et al. (2021), 95% confidence intervals were calculated for upper and lower limits of the LOAs to correct for sample uncertainty. Additionally, by dividing the difference between the upper and lower limits of the LOA into halves, a repeatability coefficient (CR) was computed that represents the largest unsystematic test-retest change to be expected (Welsch et al., 2021). Smaller CRs thus indicate larger stability.

Figure S1 visualizes the results if the Bland-Altman method. As evidenced by the black lines, the mean bias was *M* = 0.04 (*SD* = 0.40) for the eSC-IAT and *M* = -0.07 (*SD* = 0.39) for the aSC-IAT. No systematic bias was observed in terms of significant deviations from zero (*t*[109] = 1.06, *p* = .291 for the eSC-IAT and *t*[110] = -1.86, *p* = .065 for the aSC-IAT). 95% LOAs ranged from -0.75 to 0.83 for the eSC-IAT and from -0.83 to 0.69 for the aSC-IAT, implying rather large CRs of .79 for the eSC-IAT and .76 for the aSC-IAT. Absolute and relative stabilities for both SC-IATs are presented in Table S2.

Hence, no systematic bias was found, and the resulting CRs were .76 and .79 units of the SC-IAT score for both measures. This is in line with the test-retest CR of .72 for an IAT measuring sexual interest in children reported by Welsch et al. (2021). As the SC-IAT is analyzed in terms of standardized mean differences, the SC-IAT score can be interpreted in terms of Cohen’s *d* (see Welsch et al., 2021). Our results imply that a high effect size (i.e., *d* > 0.79) would be needed in order to reliably ascribe a test-retest difference to a systematic change in the implicit self-concept rather than to unreliability of the measure. In other words, moderate to high test-retest changes (*d* ≤ 0.79 and *d* ≤ 0.76, respectively) are likely to emerge because of measurement error (cf. Welsch et al., 2021).

**Study 3a**

**Participants.** 33% of the participants in the correctional/forensic sample had at least the German high-school diploma, whereas the same was true for 94% of the participants in the community sample. 65% of the final sample (*N* = 162) had at least the German high-school diploma.

**Statistical analyses.** For the PCL-R, interrater reliability was estimated using the one-way random model (ICC_1,1_) on 36 cases scored by two raters based on either the same interview or separate interviews performed by each rater. ICC for the PCL:SV was estimated based on 13 cases that were scored by two raters based on the same interview. As mean WMT-2 sum scores were strikingly low (*M* = 10.60, *SD* = 4.18), raw scores were analyzed instead of the T- or IQ-scores. 23 cases were excluded from the WMT-2 sample due to missing data in one or more items. In keeping with Karpinski and Steinman (2006), four participants were excluded due to errors in more than 20% of the trials of either the eSC-IAT or the aSC-IAT, or both. Integrity tests revealed no zero variances, no outliers in the WMT-2 or the SRP 4, and no missing data in the SRP 4. Bootstrapping (1,000 samples) was applied to compensate the violations of the normality assumption regarding PCL-R, SRP 4, and WMT-2 scores. As this does not correct for the violation of some assumptions of linear regression analyses (i.e., normality of errors, independent errors, homoscedasticity), conservative inferences should be drawn regarding H3.

Regarding H4, we had preregistered the testing of RT differences between subgroups that resembled the four clusters observed in previous studies (e.g., Krstic et al., 2018): Callous-conning, sociopathic, prototypic, and general offender/other. However, as the extrapolated PCL-R scores were positively skewed (90% of the scores below 30), the first two subsamples (callous-conning and sociopathic) were substantially smaller than the other two (prototypic and no deficit). Subgroups were therefore aggregated. The PCL factor scores were standardized, with a *z*-value above 0.67 (4^th^ quartile of the *z*-distribution) indicating an elevated score. Two independent samples *t*-tests were calculated to compare (a) participants with elevated scores on PCL-Factor 1 (i.e., callous-conning and prototypic, *n* = 46) and all other participants (i.e., sociopathic and other, *n* = 116) regarding their scores on the eSC-IAT and (b) participants with elevated scores on PCL-Factor 2 (i.e., sociopathic and prototypic, *n* = 44) and all other participants (i.e., callous-conning and other, *n* = 118) regarding their scores on the aSC-IAT. In other words, H4.1 and H4.3 as well as H4.2 and H4.4 were tested simultaneously (see online supplement for all preliminary analyses).

**Preliminary analyses.** Average psychopathy scores in the overall sample were 12.05 (*SD* = 11.36, range = 0 – 37.90, *M*_offender_ = 21.26, *SD*_offender_ = 9.31; *M*_community_ = 3.69, *SD*_community_ = 4.51) for the PCL-R and 147.74 (*SD* = 35.49, range = 86 – 250, *M*_offender_ = 169.33, *SD*_offender_ = 34.47; *M*_community_ = 128.14, *SD*_community_ = 22.84) for the SRP 4. Higher scores in the offender sample were observed for the PCL-R total score (*t*[110.19] = 15.23, *p* = .000), Factor 1 (*t*[128.68] = 11.56, *p* = .000), Factor 2 (*t*[94.87] = 16.01), the Interpersonal (*t*[150.91] = 6.31, p = .000), the Affective (*t*[111.55] = 14.55, *p* = .000), the Lifestyle (*t*[113.59] =13.68, *p* = .000), and the Antisocial facet (*t*[85.21] = 13.72, *p* = .000), the SRP total (*t*[133.35] = 8.98, *p* = .000), Factor 1 (*t*[151.79] = 3.82, *p* = .000), Factor 2 (*t*[119.27] = 12.42, *p* = .000), the Affective (*t*[147.74] = 5.03, *p* = .000), the Lifestyle (*t*[138.82] = 4.88, *p* = .000), and the Antisocial facet (*t*[103.26] = 18.13, *p* = .000), but not for the SRP 4 Interpersonal facet (*t*[164] = 1.93, *p* = .055).

Men had higher PCL-R total scores (*t*[163.41] = 10.78, *p* = .000), and higher scores on PCL-Factor 1 (*t*[155.08] = 9.07, *p* = .000), Factor 2 (*t*[149.04] = 10.72, *p* = .000), the Interpersonal (*t*[112.89] = 5.83, *p* = .000), the Affective (*t*[161.36] = 10.39, *p* = .000), the Lifestyle (*t*[163.80] = 9.87, *p* = .000) and the Antisocial facet (*t*[141.60] = 9.48, *p* = .000), a higher SRP total score (*t*[139.05] = 8.80, *p* = .000), and a higher score on SRP 4 Factor 1 (*t*[124.26] = 6.48, *p* = .000), Factor 2 (*t*[152.87] = 9.02, *p* = .000), the Interpersonal (*t*[164] = 3.32, *p* = .001), the Affective (*t*[139.71] = 8.15, *p* = .000), the Lifestyle (*t*[164] = 4.56, *p* = .000) and the Antisocial facet (*t*[161.49] = 10.45, *p* = .000). No sex effects were observed in the eSC-IAT (*t*[160] = -0.04, *p* = .967), the aSC-IAT (*t*[160] = 1.53, *p* = .129), and the cSC-IAT (*t*[160] = -1.26, *p* = .209).

Group differences neither emerged in the eSC-IAT (*t*[138.00] = -0.74, *p* = .464), nor in the aSC-IAT (*t*[160] = -0.15, *p* = .879) or the cSC-IAT (*t*[160] = -0.42, *p* = .678). For the WMT-2 scores, both sex (*t*[94.75] = -3.53, *p* = .001) and group effects were observed (*t*[125] = -4.98, *p* = .000) with higher scores observed in females (*M* = 12.32, *SD* = 3.17 vs. *M* = 9.88, *SD* = 4.36) and in the community sample (*M* = 12.04, *SD* = 3.61 vs. *M* = 8.60, *SD* = 4.12).

Negative correlations were observed between level of education and all PCL and SRP 4 scores (*rho* ≤ -.17), whereas level of education was positively correlated with WMT-2 scores (*rho* = .48, *p* = .000). No significant correlations were observed between level of education and the eSC-IAT (*rho* = .05, *p* = .539), the aSC-IAT (*rho* = -.10, *p* = .192), and the cSC-IAT (*rho* = .05, *p* = .492). Participant’s age was significantly correlated with the SRP 4 Lifestyle facet (*r* = -.21, *p* = .009), but not with the WMT-2 scores (*r* = -.04, *p* = .702) the eSC-IAT (*r* = -.14, *p* = .093), the aSC-IAT (*r* = -.13, *p* = .114), and the cSC-IAT (*r* = .07, *p* = .386). Moreover, WMT2 scores were not significantly correlated with eSC-IAT scores (*r* = .01, *p* = .951), aSC-IAT scores (*r* = -.08, *p* = .398) and cSC-IAT scores (*r* = -.06, *p* = .535). WMT-2 scores, however, were significantly correlated with overall RTs in the eSC-IAT (*r* = -.27, *p* = .002) and the aSC-IAT (*r* = -.22, *p* = .013). In the offender sample, information on potential comorbid disorders had been gathered from institutional files. No differences were observed between participants with and without comorbid diagnoses in SC-IAT scores (*t*[74] = -1.98, *p* = .051 and *t*[74] = -0.19, *p* = .852, respectively). The number of diagnostic clusters in which comorbid diagnoses occurred was unrelated to the aSC-IAT score (*r* = .04, *p* = .727), but positively correlated with the eSC-IAT score (*r* = .26, *p* = .021). No effects of prior experience with IAT-based measures was observed in a subsample of 59 participants of the community sample (*t*[57] = 0.28, *p* = .777 and *t*[57] = -0.83, *p* = .408, respectively).

**Additional Analyses.** Correlational analyses were repeated in the different subgroups as group differences in PCL-R and SRP scores had been observed. These analyses yielded a negative correlation between the eSC-IAT and the PCL-R Lifestyle facet in the offender sample (*r* = -.22, *p* = .028, 90% Bootstrap CI [-.42, -.02]), whereas all other PCL-R scores were unrelated to the eSC-IAT in both subsamples (all *p*s > .05). The aSC-IAT was positively correlated with all the PCL-R total score (*r* = .23, *p* = .020, 90% Bootstrap CI [.06, .40]), the Lifestyle (*r* = .31, *p* = .003, 90% Bootstrap CI [.15, .48]), and the Antisocial facet (*r* = .20, *p* = .038, 90% Bootstrap CI [.02, .36]). In the community sample, no significant correlations between the aSC-IAT and the PCL scores were observed (all *p*s > .05, for further details, see Table S1). Incremental validity of the multi-method assessment was observed in the offender sample regarding the PCL-R Lifestyle facet only (∆*R*² = .17, *p* = .000), with both the aSC-IAT (*B* = 2.81 *SE B* = .85, *p* = .001) and the eSC-IAT (*B* = -1.87, *SE B* = .64, *p* = .005) as significant predictors.

In addition, all analyses were repeated with WMT-2 scores included as a covariate. In the WMT-2 subsample (*n* = 123), the eSC-IAT was neither correlated with the total score nor with the Interpersonal or the Affective facet (*p* > .05). In contrast, significant partial correlations between the aSC-IAT score and PCL-R total score (*r*_p_ = .16, *p* = .040, 90% Bootstrap CI [.01, .30]) and the PCL-R Lifestyle facet (*r*_p_ = .23, *p* = .006, 90% Bootstrap CI [.07, .36]) were observed. The partial correlation between the aSC-IAT and the PCL-R Antisocial facet was not significant (*r*_p_ = .15, *p* = .052, 90% Bootstrap CI [.01, .29]).

Furthermore, when WMT-2 scores were controlled in the regression analyses, incremental validity of the multimodal assessment was observed regarding the PCL-R total score (Δ*R*^2^ = .03, *p* = .029), the PCL-R Interpersonal (Δ*R*^2^ = .05, *p* = .038), Lifestyle (Δ*R*^2^ = .07, *p* = .002), and Antisocial facets (Δ*R*^2^ = .03, *p* = .004). No incremental validity was observed in the prediction of the PCL-R Affective facet (Δ*R*^2^ = .01, *p* = .344). No significant subtype differences were observed in eSC-IAT scores (*F*[1, 120] = 0.00, *p* = .959, η_p_^2^= .00), but in aSC-IAT scores (*F*[1, 120] = 4.44, *p* = .037, η_p_^2^= .04) when WMT-2 scores were entered as a covariate. Participants with elevated scores on Factor 2 (*M* = 0.14, *SD* = 0.36) had significantly higher scores on the aSC-IAT than the other participants (*M* = -0.01, *SD* = 0.30). These results are in accordance with previous findings (Olderbak et al., 2018) and again highlight the need to control for general mental ability when using tasks that require emotion recognition.

In addition, correlational analyses between the eSC-IAT and the PCL-R scores were repeated in the offender sample with the number of clusters of comorbid diagnoses as a covariate. The results were similar to the bivariate correlation analyses (all *p*s > .05). In a series of regression analyses, the predictive validity of a combination of both SC-IATs in the prediction of the PCL-R total and facet scores was tested. The combination of both indirect measures significantly predicted the score on the PCL-R Lifestyle facet (Adj. *R*² = .03, *p* = .032), but none of the other scores (all *p*s > .05). Finally, difference scores were calculated for the average error-rates in the incompatible and the compatible block of each SC-IAT (no division by the SD due to several cases of zero intraindividual variance). These scores were unrelated to the PCL-R total score and all facet scores (all *p*s > .05).

**Study 3b**

**Participants.** 36% of the original sample had at least the German high-school diploma, whereas the same applied to 39% of the final sample for the analysis of H1 and H3 and 37% of the final sample for the analysis of H2.

**Statistical analyses.** In sum, eight participants were excluded because of elevated error-rates in the SC-IATs in either Study 1 or 2 (*n* = 6) or because of response bias in the ANTIQUE (*n* = 2). Another participant was excluded from the analyses regarding H2 owing to errors in 94% of the trials of the eCT. No missings and no outliers were found in the final subsamples. All correlational hypotheses were tested using Pearson’s correlation (H1, H3-H5) or Spearman’s rank correlation coefficients (H5.5). Hierarchical linear regression analyses were conducted in order to test the incremental validity of the eSC-IAT above and beyond the eCT (H2.1) and of the aSC-IAT above and beyond the aCT (H2.2). Again, regression analyses were conducted even though not all assumptions were met (i.e., violations regarding normality and independence of errors, homoscedasticity). Alpha-error probability was calculated one-tailed for H1 and H3 (positive correlations expected) or two-tailed for H2, H4, and H5 (significant results expected). Violations of the normality assumption were corrected using the Bootstrapping approach (90% CIs for directional, and 95% CIs for non-directional hypotheses).

**Preliminary analyses.** At t2, psychopathy scores were comparable to the scores at t1, with a mean SRP 4 score of 173.09 (*SD* = 32.43, *d*_t2-t1_ = 0.11). Level of education was neither correlated with the eSC-IAT (*rho* = -.10, *p* = .534), nor with the aSC-IAT (*rho* = -.01, *p* = .942), the eCT (*rho* = .11, *p* = .531) or the aCT (*rho* = -.11, *p* = .527). In contrast, negative correlations were found between level of education and the SRP 4 total score (*rho* = -.41, *p* = .006), the Affective (rho = -.38, p = .013) and the Antisocial facet (*rho* = -.45, *p* = .003), the UPPS Urgency (*rho* = -.33, *p* = .030) and Perseverance scales (*rho* = -.34, *p* = .027). For participant’s age, no significant correlations with the eSC-IAT (*r* = .03, *p* = .865, 95% Bootstrap CI [-.27, .33]), the aSC-IAT (*r* = -.23, *p* = .153, 95% Bootstrap CI [-.52, .08]), the eCT (*r* = .06, *p* = .725, 95% Bootstrap CI [-.27, .35]) and the aCT (*r* = .32, *p* = .051, 95% Bootstrap CI [.07, .51]) were found.

Significant correlations were observed between age and the SRP 4 total score (*r* = -.34, *p* = .027, 95% Bootstrap CI [-.58, -.01]) and the Lifestyle facet (*r* = -.56, *p* = .002, 95% Bootstrap CI [-.66, -.16]), HEXACO Conscientiousness (*r* = .34, *p* = .024, 95% Bootstrap CI [.12, .55]) and Honesty-Humility (*r* = .45, *p* = .003, 95% Bootstrap CI [.14, .66]), UPPS-P Urgency (*r* = -.30, *p* = .050, 95% Bootstrap CI [-.60, .14]), and UPPS-P Sensation Seeking (*r* = -.31, *p* = .044, 95% Bootstrap CI [-.58, .04]). For the other self-report and indirect measures, no significant correlations with age and level of education were found (all *p*s > .05).

WMT-2 scores were unrelated to the eSC-IAT score (*r* = -.17, *p* = .333, 95% Bootstrap CI [-.48, .19]), the aSC-IAT score (*r* = .06, *p* = .746, 95% Bootstrap CI [-.25, .35]), the eCT score (*r* = -.10, *p* = .574, 95% Bootstrap CI [-.41, .23]), and the aCT score (*r* = -.04, *p* = .821, 95% Bootstrap CI [-.39, .29]). Again, WMT-2 scores were negatively correlated with overall RTs in the eSC-IAT (*r* = -.47, *p* = .004, 95% Bootstrap CI [-.67, -.20]) and the aSC-IAT (*r* = -.44, *p* = .007, 95% Bootstrap CI [-.65, -.18]). However, WMT-2 scores were unrelated to overall RTs in the eCT (*r* = -.08, p = .669, 95% Bootstrap CI [-.46, .30]) and the aCT (*r* = -.01, *p* = .968, 95% Bootstrap CI [-.47, .37]). Participants with comorbid diagnoses did not differ from those without regarding either the eSC-IAT score (*t*[37] = .00, *p* = .997) the aSC-IAT (*t*[37] = 1.24, *p* = .223), the eCT (*t*[36] = -1.72, *p* = .094), or the aCT (*t*[36] = 0.61, *p* = .549), and the versatility the diagnoses was unrelated to the eSC-IAT score (*r* = .31, *p* = .056, 95% Bootstrap CI [-.12, .61]), the aSC-IAT score (*r* = -.15, *p* = .352, 95% Bootstrap CI [-.40, .13]), the eCT score (*r* = .22, *p* = .188, 95% Bootstrap CI [-.12, .52]), and the aCT score (*r* = -.21, *p* = .203, 95% Bootstrap CI [-.51, .11]).

**Additional Analyses.** Apart from the SC-IATs, test-retest correlations were also calculated for the SRP 4, yielding strong correlations between the scores at t_1_ and t_2_ (*r* = .91, *p* = .000, 95% Bootstrap CI [.83, .96]). Moreover, unexpected correlations were observed between the eSC-IAT score and the ANTIQUE (*rho* = -.36, *p* = .025), between the aSC-IAT score and HEXACO Emotionality (*r* = -.39, *p* = .014), and between the aSC-IAT and the SRP 4 Affective scale (*r* = .32, *p* = .049; see Table S3). Based on the results of Study 1, five separate regression analyses were conducted in order to test for the incremental validity of a combination of both SC-IATs and the SRP 4 above and beyond the SRP 4 alone in the prediction of PCL-R scores at t1. This time, the combination of indirect and direct measures was not incremental (all *p*s > .05). Moreover, the analyses conducted in Study 2 were again repeated with WMT-2 scores and the number of clusters of comorbid diagnoses as covariates. When WMT-2 scores were partialled out, test-retest correlations were similar for both SC-IATs, and the correlations between both SC-IATs and all external validation criteria remained non-significant (|*r*| ≤ .29, all *p*s > .05, *df* = 33).

Partial correlations with comorbidities were again only calculated for the eSC-IAT (see Study 1). These analyses revealed significant partial correlations between the eSC-IAT and the SRP 4 Lifestyle facet (*r*_p_ = -.36, *p* = .025, 95% bootstrap CI [-.58, -.12]), whereas all other relationships remained non-significant, including the test-retest correlation (|*r*| ≤ .26, all ps > .05, *df* = 36). The results of the regression analyses were also unaffected by the inclusion of either WMT 2 scores or comorbidities. Furthermore, test-retest correlations remained non-significant when the t_1_ – t_2_ time difference was controlled for (*r*_tt_ = .19, *p* = .125, 90% Bootstrap CI [-.11, .48] and *r*_tt_ = .14, *p* = .202, 90% Bootstrap CI [-.11, .38]; *df* = 36). Again, difference scores based on average error-rates were unrelated to the PCL total score and all facet scores measured at t_1_ (all *p*s > .05).

**References**

Aust, F., Diedenhofen, B., Ullrich, S., & Musch, J. (2013). Seriousness checks are useful to improve data validity in online research. *Behavior Research Methods, 45*, 527–535. <https://doi.org/10.3758/s13428-012-0265-2>

Bland, J. M., & Altman, D. G. (1986). Statistical methods for assessing agreement between two methods of clinical measurement. *The Lancet*, *327*(8476), 307–310. <https://doi.org/10.1016/S0140-6736(86)90837-8>

Bland, J. M., & Altman, D. G. (1999). Measuring agreement in method comparison studies. *Statistical Methods in Medical Research*, *8*(2), 135–160. <https://doi.org/10.1177/096228029900800204>

Chevance, G., Héraud, N., Guerrieri, A., Rebar, A., & Boiché, J. (2017). Measuring implicit attitudes toward physical activity and sedentary behaviors: Test-retest reliability of three scoring algorithms of the Implicit Association Test and Single Category-Implicit Association Test. *Psychology of Sport and Exercise*, *31*, 70–78. <https://doi.org/10.1016/j.psychsport.2017.04.007>

Glenn, A. L., & Raine, A. (2014). *Psychopathy: An introduction to biological findings and their implications.* New York University Press.

Hyde, A., Elavski, S., Doerksen, S. E., & Conroy, D. E. (2012). The stability of automatic evaluations of physical activity and their relations with physical activity. *Journal of Sport & Exercise Psychology*, *34*, 715–736. <https://doi.org/10.1123/jsep.34.6.715>

Nentjes, L., Bernstein, D. P., Cima, M., & Wiers, R. W. (2017). Implicit vs. explicit dimensions of guilt and dominance in criminal psychopathy. *International Journal of Law and Psychiatry*, *52*, 35–43. <https://doi.org/10.1016/j.ijlp.2017.03.006>

Olderbak, S. G., Mokros, A., Nitschke, J., Habermeyer, E., & Wilhelm, O. (2018). Psychopathic men: Deficits in general mental ability, not emotion perception. *Journal of Abnormal Psychology*, *127*(3), 294–304. <https://doi.org/10.1037/abn0000340>

Stieger, S., Göritz, A. S., & Burger, C. (2010). Personalizing the IAT and the SC-IAT: Impact of idiographic stimulus selection in the measurement of implicit anxiety. *Personality and Individual Differences*, *48*(8), 940–944. <https://doi.org/10.1016/j.paid.2010.02.027>

Welsch, R., Schmidt, A. F., Turner, D., & Rettenberger, M. (2021). Test-retest reliability and temporal agreement of direct and indirect sexual interest measures. *Sexual Abuse*, *33*(3), 339-360. <https://doi.org/10.1177%2F1079063220904354>
